# *Annona crassiflora* seed extract modulates histological and molecular markers associated with prostate cancer progression in TRAMP mice

**DOI:** 10.1007/s10735-026-10948-3

**Published:** 2026-09-01

**Authors:** M. J. Macedo, I. L. Lemos, I. M. U. Rossetto, F. R. Santos, F. Montico, V. H. A. Cagnon, M. R. J. Maróstica

**Affiliations:** 1 School of Food Engineering, University of Campinas (UNICAMP), Campinas, SP Brazil; 2 Department of Structural and Functional Biology, University of Campinas (UNICAMP), Campinas, SP Brazil

**Keywords:** Acetogenins, Ventral lobe, Bioactive compounds, Antiproliferative

## Abstract

Prostate cancer ranks second among the most common malignant neoplasms in men worldwide. Fruit extracts with potential anti-prostate cancer properties have gained increasing attention, with the most notable belonging to the *Annonaceae* family, including Araticum (*Annona crassiflora Mart*). In this study, we aimed to evaluate the chemopreventive and anti-inflammatory potential of Araticum seed extract (*Annona crassiflora Mart*) on the ventral prostate lobe of Transgenic Adenocarcinoma of the Mouse Prostate (TRAMP) model. Sixty male TRAMP mice, aged 8 and 12 weeks, were divided into four groups: two groups received the extract at a dose of 100 mg/kg body weight, and two control groups received equivalent volumes of water and DMSO (Dimethyl Sulfoxide). After 30 days, animals were euthanized and ventral prostate samples were collected for morphological evaluation by light microscopy, immunohistochemistry, and Western blotting. Treatment with Araticum seed extract delayed the progression of premalignant and malignant lesions (HGPIN and adenocarcinoma) and preserved healthy tissue. The extract showed antiproliferative activity, reducing PCNA, IGFR-1, and AR protein levels. Inflammatory signaling was suppressed via Toll-like receptor pathways, with decreased NFκB, TNF-α, and other inflammatory markers. Moreover, the extract reduced BCL-2 expression and promoted caspase activation, suggesting pro-apoptotic protection. In conclusion, treatment with Araticum seed extract demonstrated promising chemopreventive activity, effectively reducing the progression of epithelial lesions in the ventral prostate lobe of TRAMP mice and may be suggested as a potential chemopreventive agent in combined prostate cancer therapies.

**Graphical abstract:**

**Figure:**
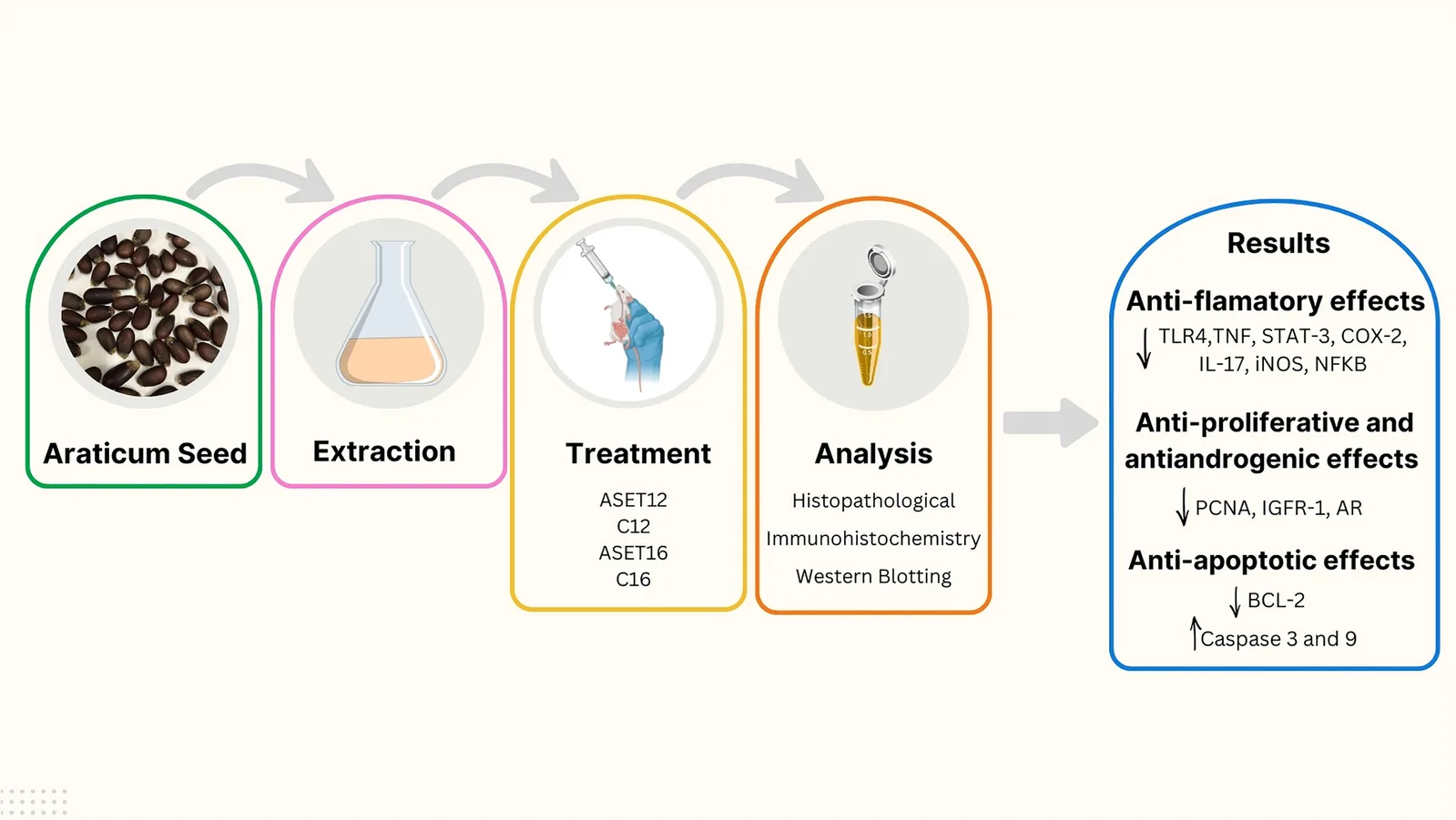

## Introduction

Prostate cancer is one of the most relevant global health issues due to its increasing prevalence, mortality rate, and the high cost of treatment (Saka et al. 2025). It is the second most common malignant neoplasm and the fifth leading cause of death among men, accounting for 1.466.680 new cases and 396.792 deaths worldwide in 2022 (Bray et al. 2024; Saka et al. 2025)^.^ Higher mortality rates are observed in about 52 countries, including regions in the Caribbean, Sub-Saharan Africa, Central and South America, and Europe (Bray et al. 2024). In Brazil, approximately 71.730 new prostate cancer cases are estimated between 2023 and 2025 (Santos et al. 2023). Prostate cancer is considered a progressive disease, characterized by continuous growth of the prostate, typically affecting men aged 45–70 years (Shorning et al. 2020; Bray et al. 2024; Saka et al. 2025). Besides age, risk factors include family history, Black ethnicity, diet, and lifestyle (Ferreira 2011; Silva 2021; Saka et al. 2025).

The prostate is a small gland located below the bladder and is part of the male reproductive system. Its main function is secretory, producing prostatic fluid that protects and nourishes sperm, aiding in their activation and capacitation. Thus, the prostate plays a significant role in male fertility (Sharma et al. 2025). Additionally, it is involved in immune functions, containing lymphocytes, macrophages, and mast cells that help combat prostate infections (Dulińska-Litewka et al. 2022). Although inflammation is essential for tissue repair, chronic inflammation in the prostate is strongly associated with increased risk for the development and progression of prostate cancer due to changes in the cellular microenvironment that favor tumor growth (Stark et al. 2015). Glandular cell genes can mutate, leading to abnormal proliferation of these cells and the formation of tumors, which may be benign (benign prostatic hyperplasia) or malignant (prostate cancer) (Salehi et al. 2019; Dulińska-Litewka et al. 2022).

Early detection of prostate cancer is important to reduce morbidity and prevent mortality (Salehi et al. 2019). Conventional chemical agents have shown various side effects, such as urinary incontinence, erectile dysfunction, loss of libido, intestinal problems, breast changes, hot flashes, and fatigue (Noh et al. 2019). Therefore, new treatment methods are needed. Dietary agents, especially foods rich in bioactive compounds such as antioxidants and phytochemicals, have shown chemopreventive properties aimed at attenuating and/or blocking primary cancer-initiating agents, with milder toxicity profiles (Salehi et al. 2019). Currently, more than 350.000 plant species have been documented worldwide, and over 25,000 phytochemicals have been identified with potent anticancer activity (Naeem et al. 2022). Approximately 62% of marketed anticancer drugs are composed of natural products or their derivatives (Newman and Cragg 2020).

**Fig. 1:**
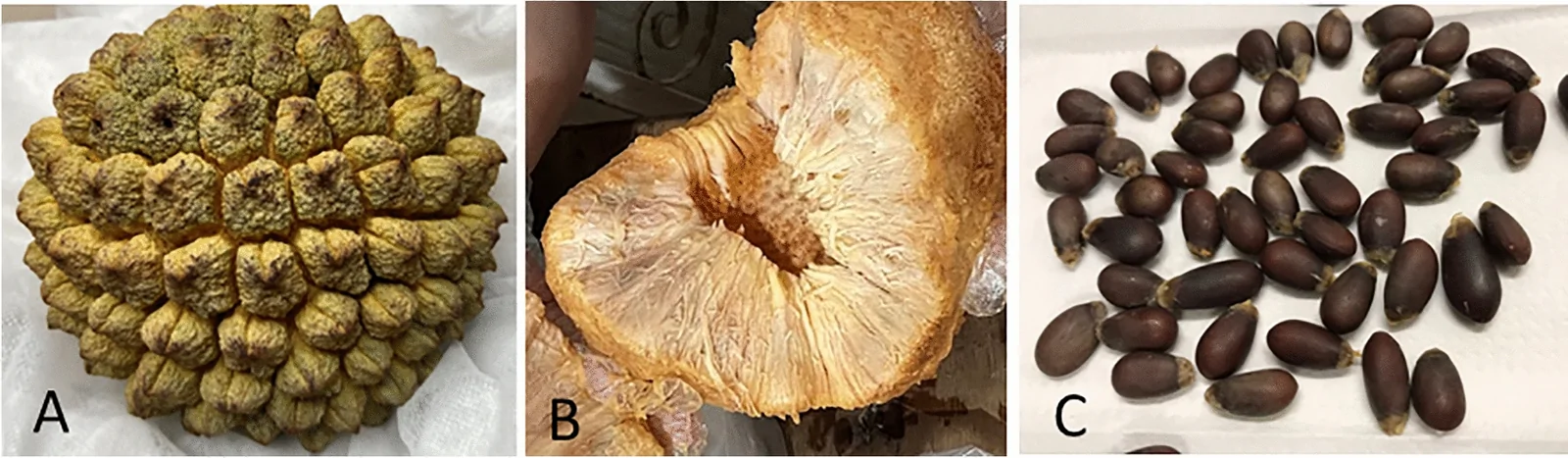
Annona *crassiflora Mart*. **A** Fruit; **B** Pulp; **C**: Seeds. Photos taken by the author

Given this, extracts from the *Annonaceae* family have gained attention for their anti-prostate cancer potential, including *Annona muricata* (soursop) and *Annona crassiflora Mart* (Araticum) (Bailão et al. 2015; Arruda & Pastore 2019). Araticum (*Annona crassiflora Mart*), native to the Brazilian Cerrado and a member of the *Annonaceae* family (Arruda 2015; Prado et al. 2020), has an oval and rounded shape, a tomentose brown skin when ripe (Fig. 1A), and fruit dimensions ranging from 9 to 15 cm in length and 10 to 15 cm in diameter. It weighs between 500 g and 4.5 kg, with a density of 1.09 g/cm^3^. On average, the fruit yield is 40% peel, 51% pulp, and 9% seeds per 100 g. The pulp is slightly sweet, has a pleasant aroma, and ranges in color from white to yellow (Fig. 1B). The seeds are dark brown, obovoid-flattened in shape, measuring 10–13 mm by 20–27 mm (Fig. 1C) (Arruda 2015).

The fruit contains a diverse array of phytochemical compounds such as carotenoids, phenolic compounds, tocopherols, as well as some vitamins and minerals (Dragano et al. 2010). Araticum seeds contain acetogenins, saturated, monounsaturated, and polyunsaturated fatty acids, phytosterols, tocopherols, and carotenoids (Arruda and Pastore 2019). Annonaceous acetogenins are a unique class of secondary metabolites from *Annonaceae* plants that exhibit anticancer, cytotoxic, antiparasitic, insecticidal, and immunosuppressive effects (Liaw et al. 2005). Their anticancer activity is linked to their ability to regulate the cell cycle, promote apoptosis by inhibiting various proteins, and even induce autophagy (Jacobo-Herrera et al. 2019).

Plant extracts are chemically complex, and their healing properties often depend on the interactions and proportions of their compounds. Much remains to be discovered about the impact of acetogenins on cancer (Quílez et al. 2018). Therefore, this field offers an opportunity to discover new molecules for the treatment of this complex disease.

Although in vitro studies using extracts from *Annonaceae* species, including *Annona muricata* (Yang et al. 2015; Rady et al. 2018; Nambooze et al. 2024), have demonstrated promising anticancer activity, evidence regarding the effects of *Annona crassiflora* seed extract during prostate cancer progression in vivo remains limited. The TRAMP model exhibits a well-characterized temporal progression of prostate carcinogenesis, with non-neoplastic lesions predominating before 12 weeks of age and preneoplastic and neoplastic lesions becoming progressively more frequent thereafter. This characteristic provides an appropriate experimental model for evaluating chemopreventive interventions at distinct stages of disease development. To the best of our knowledge, this is the first study to comprehensively characterize the inflammatory, proliferative, and apoptotic responses in the ventral prostate lobe of TRAMP mice following treatment with *Annona crassiflora* seed extract. Therefore, the present study aimed to investigate the chemopreventive potential of *Annona crassiflora* seed extract and to characterize the inflammatory, proliferative, and apoptotic responses in the ventral prostate lobe during different stages of prostate cancer progression in the TRAMP model.

## Materials and methods

This study was conducted at the Laboratory of Nutrition and Metabolism (LANUM), School of Food Engineering (FEA), in collaboration with the Laboratory of Reproductive Biology, Institute of Biology. All institutions mentioned are part of the University of Campinas (UNICAMP), Brazil.

### Raw material

The fruits of *Annona crassiflora Mart.* (araticum) were collected at the end of February and beginning of March in 2021, in natural areas of the Cerrado biome located in the municipality of Carbonita (17° 32′ 14″ S, 43° 1′ 27″ W, 747 m altitude), Minas Gerais, Brazil. The fruits were sanitized, manually peeled, and depulped. The seeds were freeze-dried (LP1010, Liobras, São Carlos, São Paulo, Brazil; 30 °C, 300 μm Hg for 95h), ground using a knife mill (Marconi, model MA 630/1, Piracicaba, SP, Brazil), and stored at − 20 °C until analysis.

### Extraction

The *Annona crassiflora* Mart. seed extract (ASE) was prepared according to a protocol previously developed and validated by our research group (Lemos et al. 2025). A total of 3 g of freeze-dried araticum seed powder was homogenized with 30 mL of a 75% (v/v) ethanol–water solution and extracted in a thermostatic bath (MA093 Marconi Dubnoff) at 37 °C under constant agitation for 90 min. The extract was vacuum-filtered, and the residue was subjected to a second extraction under the same conditions. The ethanolic extracts were pooled and concentrated under reduced pressure using a rotary evaporator (MA120 Marconi) to remove the ethanol. The remaining aqueous extract was subsequently freeze-dried to obtain the dry extract, which was stored at − 20 °C until use in the in vivo experiments.

Importantly, the same batch of ASE used in the present study had been previously subjected to a comprehensive phytochemical characterization by liquid chromatography coupled to tandem mass spectrometry operating in multiple reaction monitoring mode (LC-MS/MS-MRM) (Lemos et al. 2025). This analysis identified gallic acid (104.67 mg/g), catechin (97.27 mg/g), quercetin (47.6 mg/g), and rutin (43.8 mg/g) as the major phenolic constituents of the extract. In addition, the antioxidant capacity of this same extract was previously evaluated using the total phenolic content (TPC), ABTS, FRAP, and ORAC assays, yielding values of 26.49 mg GAE/g fdw, 248.5 μmol TE/g fdw, 42.4 μmol TE/g fdw, and 211.15 μmol TE/g fdw, respectively (Lemos et al. 2025). The extract was previously characterized with emphasis on its phenolic composition, which constituted the primary focus of the present biological investigation. Thus, the phytochemical composition and antioxidant properties reported in the present study correspond directly to the extract administered to the animals.

The study was registered in the Brazilian National System for the Management of Genetic Heritage and Associated Traditional Knowledge (SISGEN) under registration number AA49469.

### Animals and experimental procedure

A total of 60 male TRAMP transgenic mice (C57BL/6-Tg (TRAMP) 8247Ng/J X FVB/NJ) F1/J were used, because this transgenic model specifically develops prostate cancer through androgen-dependent expression of the SV40 T antigen in the prostate epithelium, obtained from the Multidisciplinary Center for Biological Research (CEMIB), University of Campinas (UNICAMP). All animals were housed individually in polypropylene cages with white pine wood shavings, provided with water and chow ad libitum (Nuvilab, Colombo, PR, Brazil), and maintained in the Animal Facility of the Department of Structural and Functional Biology (Anatomy Area), Institute of Biology, under controlled environmental conditions: temperature (22 °C ± 2 °C), relative humidity (55% ± 10%), 12h light/dark cycle, and continuous air exhaust, until they reached the experimental age for their respective treatment group.

The dose and treatment duration were determined based on previous data reported by our group involving the characterization and application of the extract in an in vitro prostate cancer model (Lemos et al. 2025) and an in vivo TRAMP model (Lemos et al. 2026), as well as studies investigating extracts from *Annona muricata* (Yang et al. 2015), and *A. crassiflora* in prostate cancer models (Prado et al. 2020; Lemos, et al. 2025).

The selected in vivo dose was not directly extrapolated from the in vitro concentrations. Instead, it was established through a biologically plausible and safe pilot study, where it was evaluated two doses of ASE (50 and 100 mg/kg body weight [bw]). Both doses demonstrated promising biological activity; however, the 100 mg/kg bw dose produced more efficient biological responses. The absence of treatment-related toxicity was further evaluated by histopathological examination of the liver and prostate (Figs. 4, 5 and 6). No histopathological alterations indicative of hepatic toxicity were observed in liver sections from treated animals, indicating that the selected 100 mg/kg bw dose was safe and biologically relevant for evaluating the chemopreventive effects of ASE in the TRAMP model. However, liver biochemical parameters were not evaluated in this study, and therefore systemic toxicity cannot be completely excluded.

Two experimental treatment intervals were adopted to investigate the effects of ASE at different stages of prostate carcinogenesis. The treatment duration was established according to the well-characterized temporal progression of the disease in the TRAMP model. Animals treated from 8 to 12 weeks of age were evaluated during a stage in which non-neoplastic lesions predominate, allowing the evaluation of the chemopreventive potential of ASE. In contrast, animals treated from 12 to 16 weeks of age were evaluated during disease progression, when preneoplastic and neoplastic lesions become increasingly prevalent. Thus, the 30-day treatment period allowed the investigation of ASE during both the early and progressive stages of prostate carcinogenesis while providing sufficient exposure to detect histopathological and molecular alterations.

**Fig. 2:**
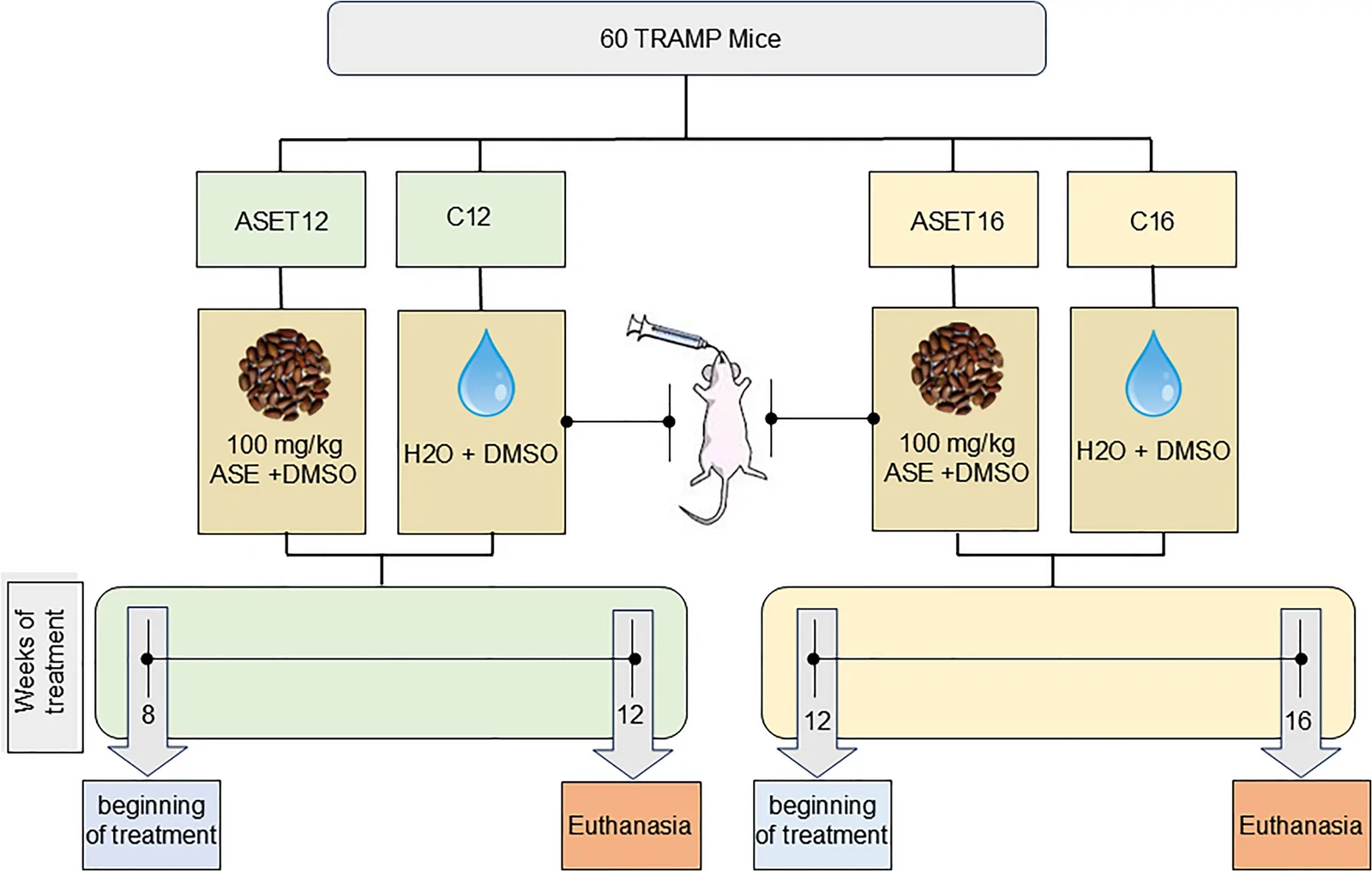
Experimental design for the division of TRAMP mice into different experimental groups, as well as the treatment periods with ASE and euthanasia of the animals

The animals were randomly divided into experimental groups as described in Fig. 2. Two experimental groups were assessed during the early stage of disease (at 8 weeks of age) and two groups during the advanced stage of lesion development (at 12 weeks of age), as follows:

- *ASET12 group (8–12 weeks):* 15 male TRAMP mice received 100 mg/kg body weight of ASE diluted in 10% DMSO, administered orally by gavage five times per week until 12 weeks of age.
- *C12 group (8–12 weeks):* 15 male TRAMP mice received water and 10% DMSO (vehicle used to dilute ASE) in volumes equivalent to the ASET12 group, administered by gavage five times per week until 12 weeks of age.
- *ASET16 group (12–16 weeks):* 15 male TRAMP mice received 100 mg/kg body weight of ASE diluted in 10% DMSO, administered by gavage five times per week until 16 weeks of age.
- *C16 group (12–16 weeks):* 15 male TRAMP mice received water and 10% DMSO in volumes equivalent to the ASET16 group, administered by gavage five times per week until 16 weeks of age.

After 30 days of treatment, all animals were weighed using a semi-analytical scale (Marte AS 5500, São Paulo, Brazil), anesthetized with 2% Xylazine Hydrochloride (5 mg/kg bw, administered intramuscularly [i.m.]; König, São Paulo, Brazil) and 10% Ketamine Hydrochloride (60 mg/kg bw, i.m.; Fort Dodge, Iowa, USA), and euthanized in accordance with the ethical committee recommendations (CEUA:5976-1/2022) for the use of experimental animals. Samples of the ventral prostate lobe were collected from animals in each experimental group and subjected to morphological analysis via light microscopy, immunohistochemistry, and western blotting.

### Histopathological analysis

Samples of the ventral prostate lobe from five animals in each experimental group were collected and fixed in Bouin’s solution for 24h. After fixation, the prostate tissues were washed in 70% ethanol and then dehydrated in a graded alcohol series. Subsequently, the tissue fragments were cleared in xylene and embedded in plastic polymers (Paraplast Plus, St. Louis, MO, USA). The materials were then sectioned at 5 µm thickness using a Hyrax M60 microtome (Zeiss, Munich, Germany). The sections were stained with Hematoxylin and Eosin and photographed using a Nikon Eclipse E-400 photomicroscope (Nikon, Tokyo, Japan).

Histological classification was performed for the different degrees of lesions found in the ventral prostate lobe of TRAMP mice, according to the modified methods of Berman-Booty et al. (2012); Montico et al. (2023), and Santos et al. (2024).

**Fig. 3:**
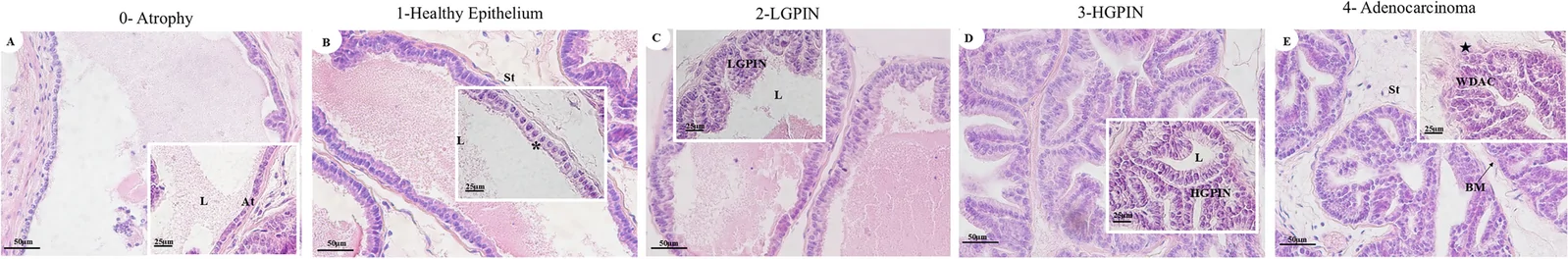
Photomicrographs of morphological parameters of the prostatic epithelium used in the histopathological analysis of the ventral prostate lobe from the experimental groups. Graded according to the presented scale: atrophy (At), healthy epithelium (*), low-grade prostatic intraepithelial neoplasia (LGPIN), high-grade prostatic intraepithelial neoplasia (HGPIN), and well-differentiated adenocarcinoma (WDAC). (★) Invasion of epithelial cells into the stroma, L: lumen; St: stroma. Staining: hematoxylin and eosin. Magnification: 40x and 100x

- *Atrophy *(Fig. 3A): Characterized by the presence of a single layer of basal cells with scarce cytoplasm and irregular nuclei.
- *Healthy epithelium *(Fig. 3B): Characterized by a monolayer of cuboidal to columnar epithelial cells with basally oriented nuclei.
- *Low-grade prostatic intraepithelial neoplasia (LGPIN) *(Fig. 3C): Characterized by epithelial cell proliferation forming different acinar organizations, classified based on how the stratified epithelium lines the lumen (wavy, flat, micropapillary). The basal membrane remains intact. Hyperplastic cells with prominent, pleomorphic, and hyperchromatic nuclei are observed.
- *High-grade prostatic intraepithelial neoplasia (HGPIN) *(Fig. 3D): Characterized by multiple layers of hyperplastic cells forming a cribriform pattern, with prominent and enlarged nuclei extending throughout the glandular acinus, potentially obstructing the luminal space partially or completely, forming small hyperplastic acini without associated stroma.
- *Well-differentiated adenocarcinoma *(Fig. 3E): Characterized by hyperplastic epithelial cells with nuclear pleomorphism (numerous, prominent nuclei of irregular sizes, and hyperchromatic staining). In this lesion, the fibromuscular tissue layer shows discontinuities, leading to the invasion of epithelial cells into the stroma.

For each animal, ten random fields were captured at 40x magnification and divided into four quadrants. In each quadrant, the predominant morphological characteristic was classified according to the following criteria: (0) atrophy (At); (1) healthy epithelium (HE); (2) low-grade prostatic intraepithelial neoplasia (LGPIN); (3) high-grade prostatic intraepithelial neoplasia (HGPIN); and (4) well-differentiated adenocarcinoma (WDAC), as described in Fig. 3. The percentage of each lesion type was then established for each experimental group (Rossetto et al. 2023; Santos et al. 2024; Rezende et al. 2025).

### Liver histopathological analysis

Samples from the livers of five animals from each experimental group were collected and prepared as described in Sect. “Histopathological analysis”. The liver histopathological analysis was done using the softwares NIS-Elements/Image and Image Pro-Plus in digital microscopic images obtained with the Nikon Eclipse E-400 microscope (Nikon, Tokyo, Japan). A grid containing 200 intersections was superimposed over 10 randomly obtained images per animal at 40× magnification, and the following parameters were quantified: mononucleate hepatocyte, binucleate hepatocyte, total hepatocyte cytoplasm, lipid hepatic content, blood vessel and inflammatory infiltrate (Kleiner et al. 2005; Takahashi & Fukusato 2014; Lamas et al. 2018).

### Immunohistochemistry

Samples from the ventral prostate lobe of five animals from each experimental group were collected to confirm the immunolocalization of target proteins in the prostate tissue. Sections (5 µm thick) were obtained using a Hyrax M60 microtome (Zeiss, Munich, Germany) and mounted on silanized slides. Antigen retrieval was performed by submerging the sections in citrate buffer, heated for 25 min at 100 °C in a microwave oven, followed by 15 min at room temperature. Endogenous peroxidase activity was blocked using hydrogen peroxide (0.1% and 0.3% in methanol) for 20 min, followed by incubation with 3% bovine serum albumin (BSA) in TBS-T buffer for 1h at room temperature.

The primary antibodies used were: iNOS (ab136918, Abcam, Cambridge, MA, USA) at a 1:50 dilution; IL-17 (ab79056, Abcam) at 1:150; and PCNA (ab29, Abcam) at 1:600, all diluted in 1% BSA and incubated overnight at 4 °C. Sections were then washed in TBS-T and incubated with HRP-conjugated goat anti-rabbit IgG secondary antibody (W4018, Promega) for 2h, or with the EnVision HRP secondary antibody (Dako Inc., USA) for 15 min. Peroxidase activity was revealed using 3,3′-diaminobenzidine (DAB, Sigma-Aldrich), and Harris hematoxylin was used for counterstaining. Negative controls were included by omitting the primary antibody.

A point-counting analysis based on Weibel (1963) multipoint system, using 850 intersection points, was performed. Ten random images per animal were captured using a Nikon Eclipse E-400 photomicroscope (Nikon, Tokyo, Japan) at 40× magnification. Quantification of positive cells was determined by the presence of brown cytoplasmic staining. Immunoreactivity coinciding with grid intersections was counted and divided by the total number of points (Weibel 1963; Mateus et al. 2019; Montico et al. 2023). Results were expressed as the relative frequency of positive labeling for each antigen across the experimental groups.

### Protein quantification by western blotting

Samples from the ventral prostate lobe of five animals per group were collected and stored at − 80 °C. Proteins were extracted using lysis buffer (150 mM NaCl, 50 mM Tris–HCl, 1% Triton, 0.1% SDS, pH 8.0) supplemented with 1% aprotinin (A6279, Sigma-Aldrich, St. Louis, MO, USA). Samples were homogenized using a SONICS Vibra-Cells™ ultrasonic sonicator (Sonics & Materials, Inc.), then centrifuged at 15,000 rpm for 20 min at 4 °C (Sigma® 3–18 K refrigerated centrifuge). Protein concentration in the supernatant was determined using the Bradford method (Bio-Rad Laboratories, CA, USA).

Protein samples were mixed 1:1 with Laemmli sample buffer (Bio-Rad) containing 5% β-mercaptoethanol and incubated at 100 °C for 5 min. A total of 50 µg of protein per sample was loaded onto a 10% SDS–polyacrylamide gel. Following electrophoresis, proteins were transferred to nitrocellulose membranes (Hoefer System) at 120 V for 120 min. Membranes were blocked with 3% BSA or 5% non-fat dry milk in TBS-T for 1h and incubated overnight with primary antibodies, followed by 2h incubation with secondary antibodies the next day (Table 1). Blots were visualized using a chemiluminescent detection kit (SuperSignal West Pico, Thermo Scientific, 34,080). Bands were imaged using the GeneGenome–Genesys system and a chemicamera (Syngene, Cambridge, UK). Pixel densitometry was quantified using Uni-Scan-It 6.1 software. β-actin was used as the endogenous loading control (Kido et al. 2022; Rossetto et al. 2023).

**Table 1:** Antibodies used for western blotting (WB) and immunohistochemistry (IHC)

Antibodies	Specifications	Dilution
Anti-mouse (secondary)	W4021- Promega corporation	1:2000–1:5000
Anti-rabbit (secondary)	W4018- Promega corporation	1:2000–1:5000
AR	ab74272- Abcam	1:500 (WB)
Β-actina	sc 81178- Santa cruz biotechnology	1:500(WB)
BCL-2	3869- Cell signaling biotechnology	1:250(WB)
Caspase 3	sc-56053- Santa cruz biotechnology	1:500(WB)
Caspase 9	sc-56076- Santa cruz biotechnology	1:500(WB)
COX-2	sc-376861-Santa cruz biotechnology	1:250 (WB)
IGFR-1	sc-712- Santa cruz biotechnology	1:350 (WB)
IL-17	ab79056- Abcam	1:150 (IHC)
iNOS	ab136918- Abcam	1:50 (IHC)
NFKB	ab 13594- Abcam	1:500(WB)
PCNA	ab-29- Abcam	1:600(IHC)/1:500 (WB)
Phospho-NfκB p65	sc-271908- Santa cruz biotechnology	1:250(WB)
Phospho-STAT3	3E2- Cell signaling technology	1:250(WB)
STAT-3	sc-8019- Santa cruz biotechnology	1:250(WB)
TLR4	sc-293072- Santa cruz biotechnology	1:250(WB)
TNF-α	ab8348- Abcam	1:2000(WB)

### Statistical analysis

Statistical analysis of prostatic lesion incidence (morphology, PCNA, immunohistochemistry, and Western blotting) among the experimental groups was considered parametric following the Shapiro–Wilk normality test, and comparisons were made using the Student’s t-test. Analyses were conducted using GraphPad Prism software (version 7.00), with the level of significance set at 5%. Results are expressed as mean ± standard deviation.

## Results

### Histopathological alterations in TRAMP mouse prostate following ASE treatment

**Fig. 4:**
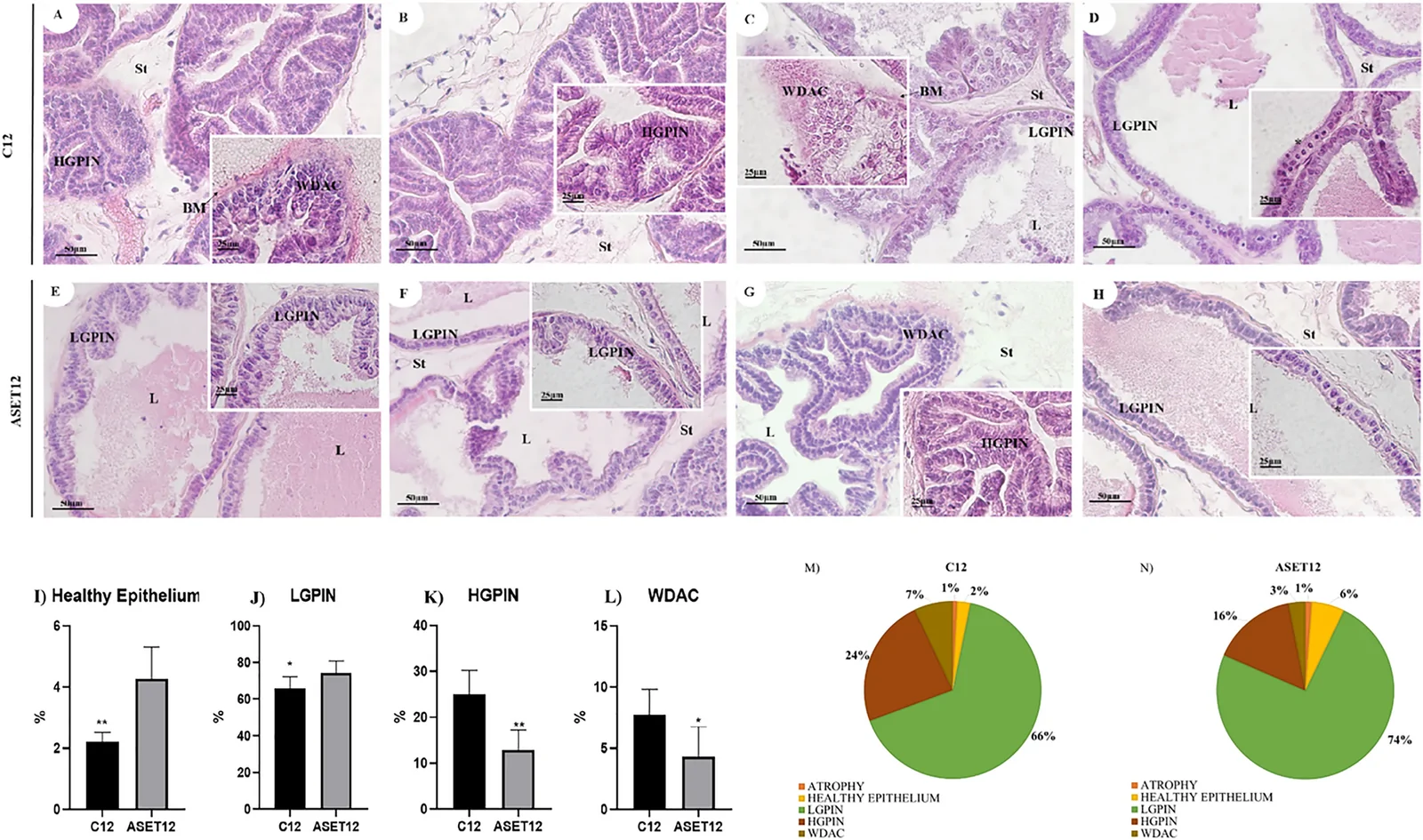
Photomicrographs of the ventral prostate lobe from TRAMP mice in groups C12 (**A**–**D**) and ASET12 (**E**–**H**). Presence of HGPIN (high-grade prostatic intraepithelial neoplasia) with cells filling the lumen in a cribriform pattern (**A**, **B**, and **G**) and a focus of WDAC (well-differentiated adenocarcinoma), showing basal membrane (BM) rupture and infiltration of epithelial cells toward the stroma (**A**, **C**, and **G**). Presence of LGPIN (low-grade prostatic intraepithelial neoplasia), characterized by hyperplastic epithelium forming papillary projections toward the lumen (**B**, **C**, **D**, **E**, **F**, and **H**). Areas with healthy epithelial morphology were identified (*), showing simple columnar secretory epithelium interspersed with basal cells (**D** and **H**). Ep: epithelium; St: stroma; L: lumen. **I**–**L** Incidence of different epithelial morphological lesions in the ventral prostate lobe of TRAMP mice in the ASET12 and C12 groups. **M**–**N** Analysis of the incidence of epithelial characteristics within each experimental group. For all analyses, statistical significance was considered between the ASE-treated group and the untreated group within the same experimental period (* = *p* < 0.05; ** =*p* < 0.01; **** = *p* < 0.0001). Staining: Hematoxylin and eosin. Magnification 40x and 100x

The ventral prostate lobe of the ASET12 group predominantly exhibited lesions classified as low-grade prostatic intraepithelial neoplasia (LGPIN), along with areas of healthy epithelium (Fig. 4J and I, respectively). In contrast, the C12 group displayed a significantly higher frequency (*p* < 0.05) of high-grade PIN (HGPIN) and well-differentiated adenocarcinoma (WDAC) (Fig. 4K and L).

Accordingly, the frequency of LGPIN was significantly higher (*p *< 0.05) in the ASET12 group (74.38%) compared to the C12 group (66.13%) (Fig. 4J). LGPIN was characterized by epithelial cell stratification extending variably into the acinar lumen, forming short or tall papillary projections with poorly defined acinar contours (Fig. 4B). Moreover, a 6% incidence of healthy epithelium was observed in the ASET12 group, versus only 2.4% (*p* < 0.01) in the C12 group (Fig. 4I). Healthy epithelium was identified by a single layer of cuboidal to columnar epithelial cells with basally oriented nuclei (Fig. 4D and H).

The frequency of HGPIN was significantly lower (*p *< 0.001) in the ASET12 group (15.38%) compared to the C12 group (25.58%) (Fig. 4K). HGPIN lesions exhibited hyperplastic epithelial cells projecting into the lumen with a cribriform architecture, a marked increase in nuclear-to-cytoplasmic ratio, and prominent nucleoli (Fig. 4B and G). The occurrence of well-differentiated adenocarcinoma was also significantly reduced (*p *< 0.05) in the ASET12 group (3.08%) relative to the C12 group (7.06%) (Fig. 4L). These lesions were characterized by nuclear pleomorphism, defined by markedly irregular nuclear sizes, prominent and numerous nucleoli, and evidence of stromal invasion (Fig. 4A and C).

**Fig. 5:**
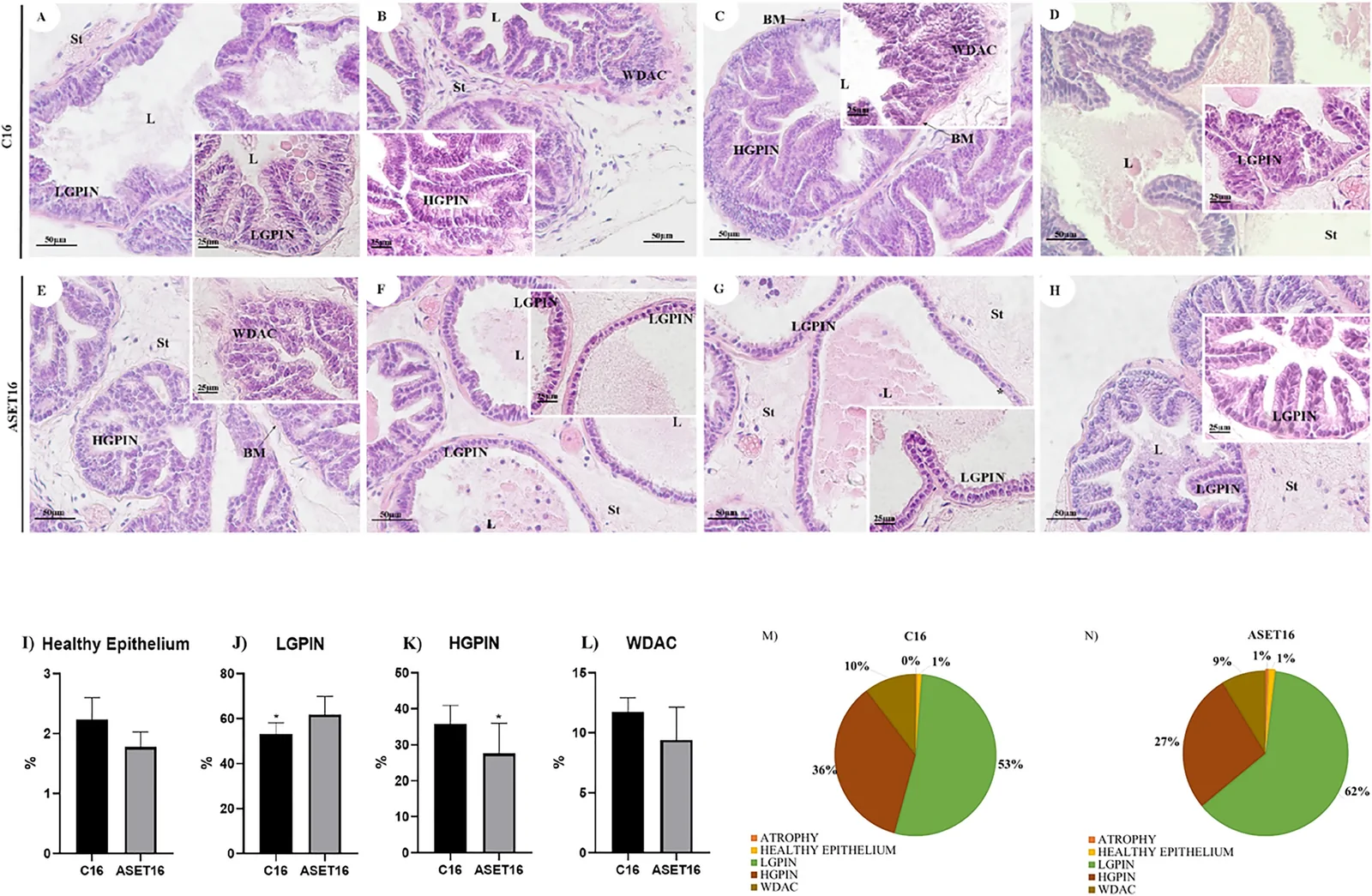
Photomicrographs of the ventral prostate lobe of TRAMP mice from the C16 (**A**–**D**) and ASET16 (**E**–**H**) groups. Presence of LGPIN (low-grade prostatic intraepithelial neoplasia), characterized by hyperplastic epithelium forming papillary projections toward the lumen (**A**, **D**, **F**, **G**, and **H**). Presence of HGPIN (high-grade prostatic intraepithelial neoplasia) with cells filling the lumen in a cribriform pattern (**B**, **C**, and **E**), and focus of WDAC (well-differentiated adenocarcinoma) showing basement membrane (BM) disruption and epithelial cell infiltration into the stroma (**B**, **C**, and **E**). Areas with healthy epithelial morphology (*) are characterized by a simple columnar secretory epithelium interspersed with basal cells (**G**). Ep: epithelium; St: stroma; L: lumen. **I**–**L** Incidence of different epithelial morphological lesions in the ventral prostate lobe of TRAMP mice from the ASET16 and C16 groups. **M**–**N** Incidence analysis of epithelial characteristics within each experimental group. For all analyses, statistical significance was considered between ASE-treated and untreated groups within the same experimental period (= *p *< 0.05; *** = *p *< 0.001). Staining: Hematoxylin and eosin. Magnification 40x and 100x

In the ventral prostate lobe of the ASET16 group, lesions were predominantly characterized as LGPIN and the presence of healthy epithelium (Fig. 5J and I, respectively), whereas in the C16 group there was a significantly higher frequency (p<0.05) of HGPIN as well as the presence of WDAC (Fig. 5K and L).

Accordingly, a 61.78% frequency of LGPIN (*p* < 0.05) (Fig. 5. F, G, and H) was observed in the ASET16 group, compared to approximately 53.06% in the C16 group (Fig. 5J). Additionally, in the ASET16 group, a higher frequency of healthy epithelium (Fig. 5G) was noted (1.36%), while in the C16 group only 0.96% was observed (Fig. 5I). The incidence of HGPIN (Fig. 5 B and E) was lower (*p* < 0.05) in the ASET16 group (27.61%) (Fig. 5K) compared to the C16 group (35.46%). A similar pattern was observed for well-differentiated adenocarcinoma lesions (Fig. 5B and C), with lower frequency in the ASET16 group (8.71%) versus the C16 group (10.39%) (Fig. 5L).

**Fig. 6:**
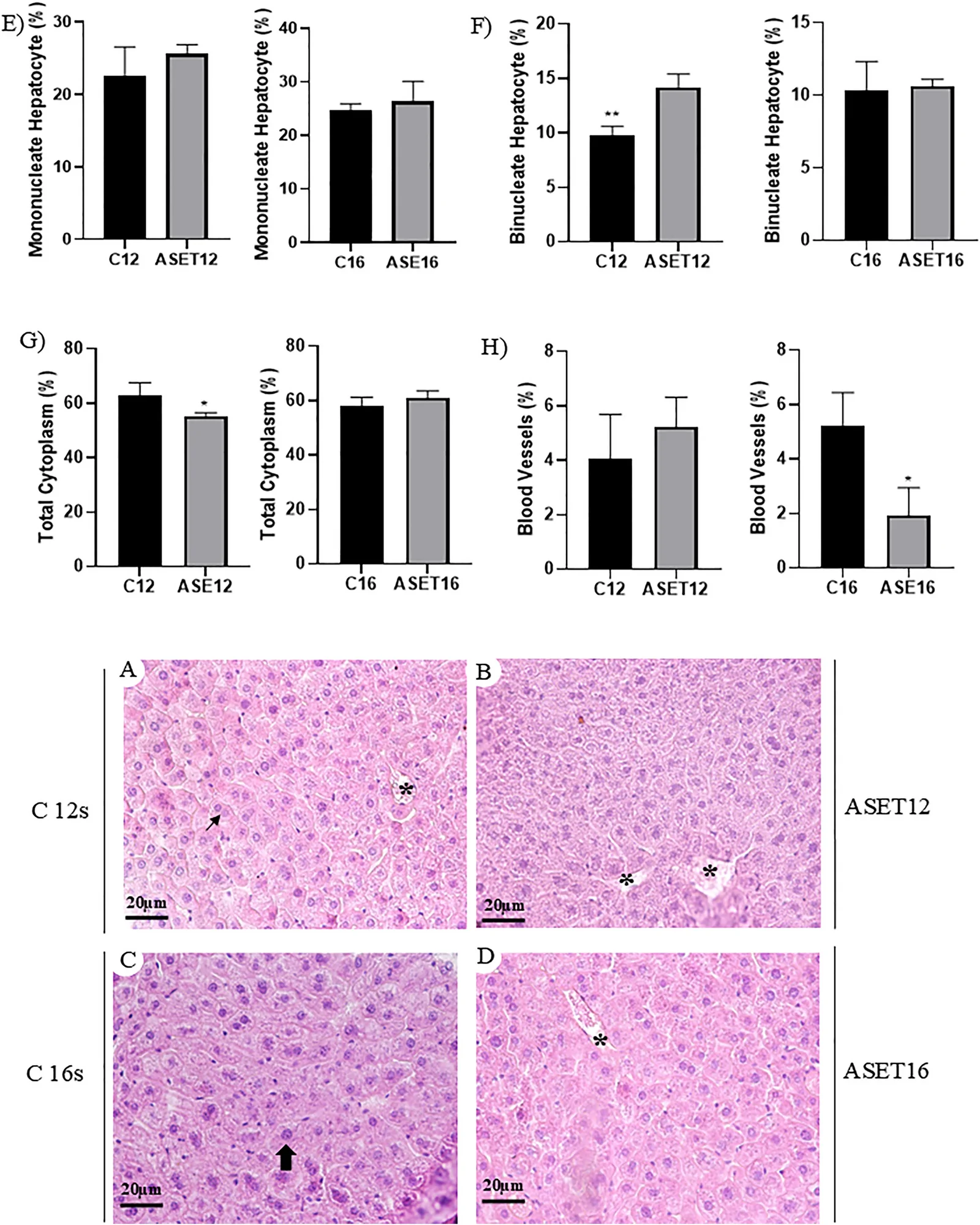
Photomicrographs of the liver of TRAMP mice from groups C12 (**A**), ASET12 (**B**), C16 (**C**), ASET16 (**D**), showing binucleate hepatocyte (thin arrow); mononucleate hepatocyte (thick arrow); blood vessels (*), and Histopathological analysis: **E** Mononucleate Hepatocyte (%), **F** Binucleate Hepatocyte (%), **G** Total Cytoplasm (%), **H** Blood Vessels (%). Incidence analysis of epithelial characteristics within each experimental group. For all analyses, statistical significance was considered between ASE-treated and untreated groups within the same experimental period (* = *p* < 0.05; ** = *p *< 0.01). Staining: Hematoxylin and eosin. Magnification 40x.

These findings are consistent with the results observed in the immunohistochemical analysis and protein quantification by Western blotting. A decrease in cell proliferation was observed following ASE treatment, with a significant reduction in epithelial immunolocalization of the proliferation marker PCNA in the ASET12 and ASET16 groups (Fig. 6E and F). Additionally, at both treatment time points (12 and 16 weeks), PCNA protein levels (Fig. 6G and H) significantly decreased after treatment (**p < 0.01, ***p < 0.001).

### Histopathological features of the liver in TRAMP mice treated with ASE

The liver morphology of the ASET12 and ASET16 group, compared to their respective control groups (C12 and C16), showed a typical liver morphology and, therefore, was scored as 0 regarding the Nonalcoholic fatty liver disease (NAFLD) (Kleiner et al. 2005; Takahashi and Fukusato 2014). As shown in Fig. 6A and C, the hepatocytes displayed a polygonal format with 1–2 central nuclei (mononuclear or binuclear) they are grouped into cords that anastomose with each other (Gerra 2006; Lamas et al. 2018). Although significant differences were observed in blood vessel and cytoplasmic morphology, these changes were more pronounced in the control groups (cytoplasm at C12 weeks and blood vessels at C16 weeks) than in the extract-treated groups ESA.

Furthermore, the higher number of binucleated hepatocytes observed in the treated group at ASE12 is not necessarily indicative of liver injury. Binucleated hepatocytes are a physiological characteristic of the adult mammalian liver resulting from hepatocyte polyploidization and are considered an important component of liver homeostasis, metabolic adaptation, and regenerative capacity (Duncan & Soto-Gutierrez 2013; Wang et al. 2017; Donne et al. 2020). Therefore, no histopathological features consistent with hepatic steatosis or other overt liver injury, such as inflammatory infiltrates, hepatocellular necrosis, or tissue disorganization, were detected. Therefore, these isolated morphological changes were not considered indicative of hepatotoxicity treatment with ESA compared to their respective control groups (Fig. 6E–H).

### Decreased PCNA expression shows the antiproliferative action of ASE in TRAMP mice

**Fig. 7:**
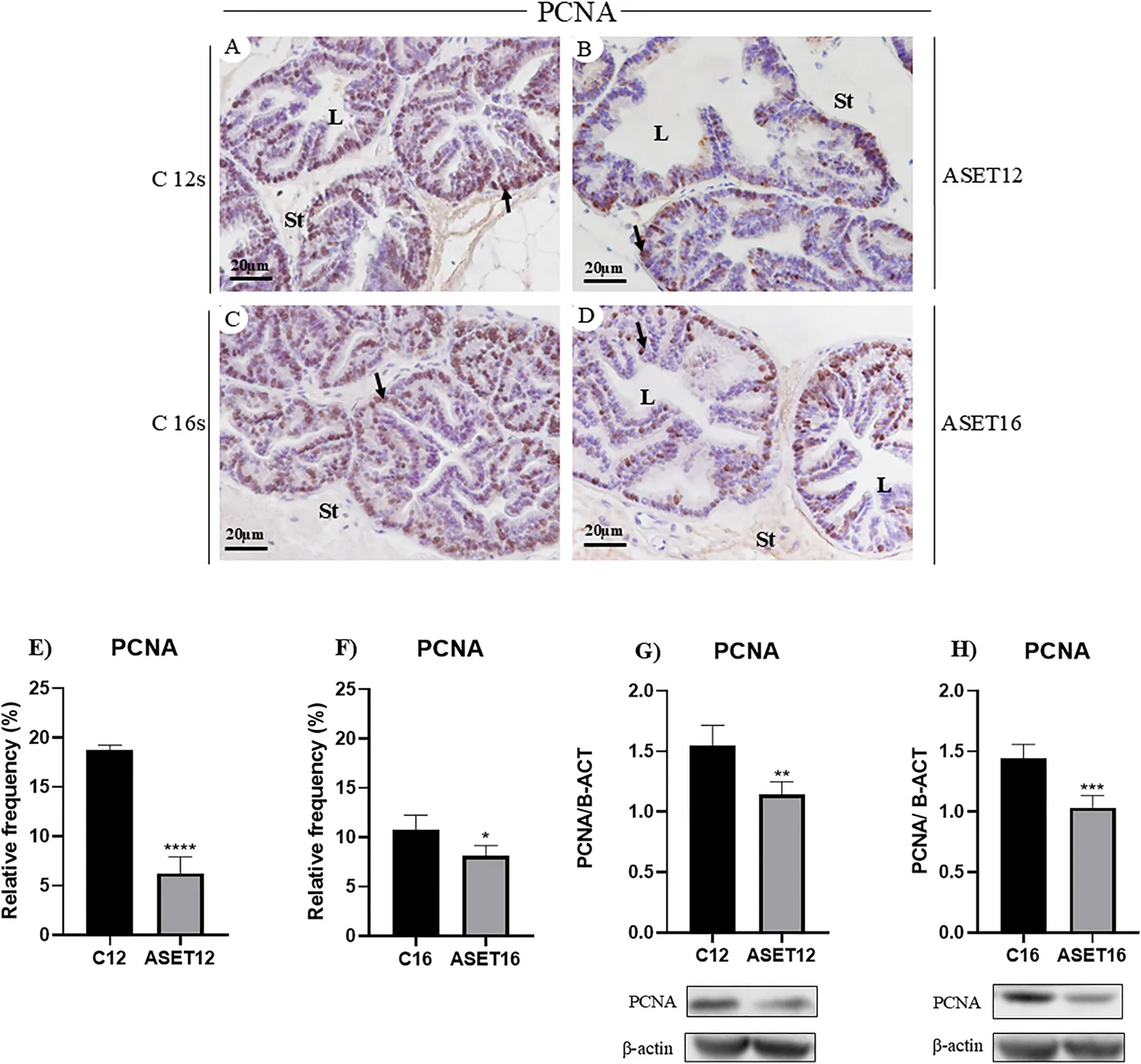
Photomicrographs of the ventral prostate lobe of TRAMP mice from groups C12 (**A**), ASET12 (**B**), C16 (**C**), ASET16 (**D**), showing PCNA immunolabeling in epithelial cells (black arrows). St: stroma; L: lumen. **E** and **F** Frequency of PCNA immunolabeling in the ventral prostate lobe of TRAMP mice from the ASET12 and ASET16 groups and their respective controls. **G** and **H** Protein levels of PCNA in the ventral prostate lobe of TRAMP mice from the ASET12 and ASET16 groups and their respective controls. For all analyses, statistical significance was considered between the ASE-treated group and the untreated group within the same experimental period (* = *p* < 0.05; ** = *p* < 0.01; *** = *p* < 0.001; **** = *p* < 0.0001). Counterstaining with hematoxylin. 40x magnification

In the ventral prostate lobe, a significant decrease in cell proliferation was observed in the ASET12 and ASET16 groups (*p* < 0.05, *p *< 0.0001), based on nuclear immunolocalization of the proliferation marker PCNA (Fig. 7A–F), compared to their respective control groups (C12 and C16). These findings are consistent with results from Western blotting protein quantification analysis, where in both treatment durations (12 and 16 weeks), PCNA protein levels significantly decreased following ASE treatment (Fig. 7G and H).

### Downregulation of inflammatory markers by Araticum seed extract (ASE)

#### Reduction of inducible nitric oxide synthase (iNOS) and interleukin-17 pathways

**Fig. 8:**
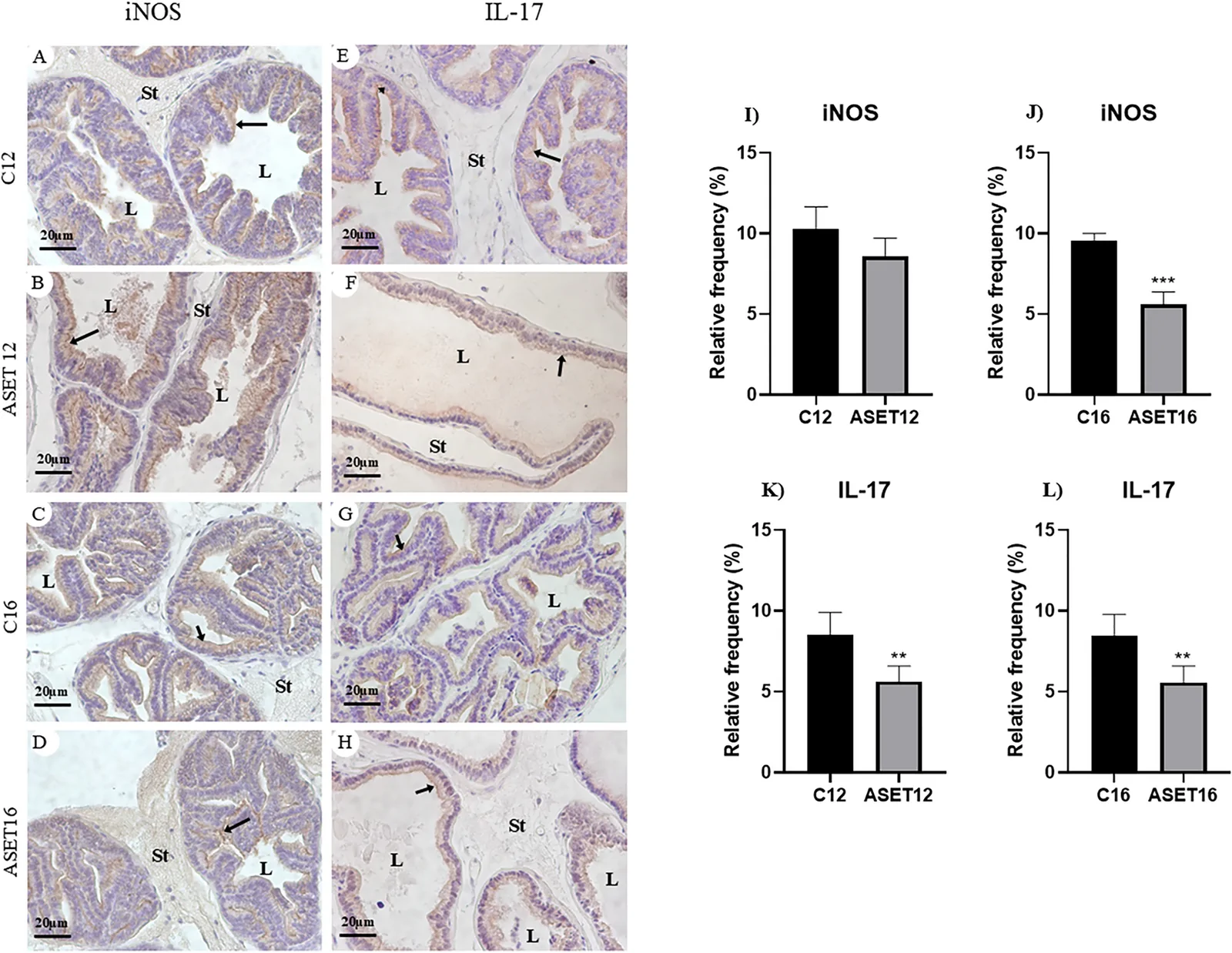
Photomicrographs of the ventral prostate lobe of TRAMP mice from the C12 (**A** and **E**), ASET12 (**B** and **F**), C16 (**C** and **G**), and ASET16 (**D** and **H**) groups, showing iNOS and IL-17 immunolabeling in epithelial cells (black arrows). St: stroma; L: lumen. (**I** and **J**) Frequency of iNOS immunolabeling in the ventral prostate lobe of TRAMP mice from the ASET12 and ASET16 groups and their respective controls. **K** and **L** Frequency of IL-17 immunolabeling in the ventral prostate lobe of TRAMP mice from the ASET12 and ASET16 groups and their respective controls. For all analyses, statistical significance was considered between ASE-treated and untreated groups within the same experimental period (** = *p *< 0.01; *** = *p* < 0.001). Counterstaining with hematoxylin. 40x magnification

In the ventral prostate lobe, a significant decrease (*p* < 0.001; *p *< 0.01) in the cytoplasmic immunohistochemical labeling of inducible nitric oxide synthase (iNOS) and interleukin-17 (IL-17) was observed in the ASET16 group compared to the C16 group (Fig. 8J and L). However, in the ASET12 group, a significant decrease (*p* < 0.001) was observed only for IL-17 compared to the C12 control group (Fig. 8K).

#### Reduction of inflammation and cell proliferation through downregulation of the STAT-3 pathway

**Fig. 9:**
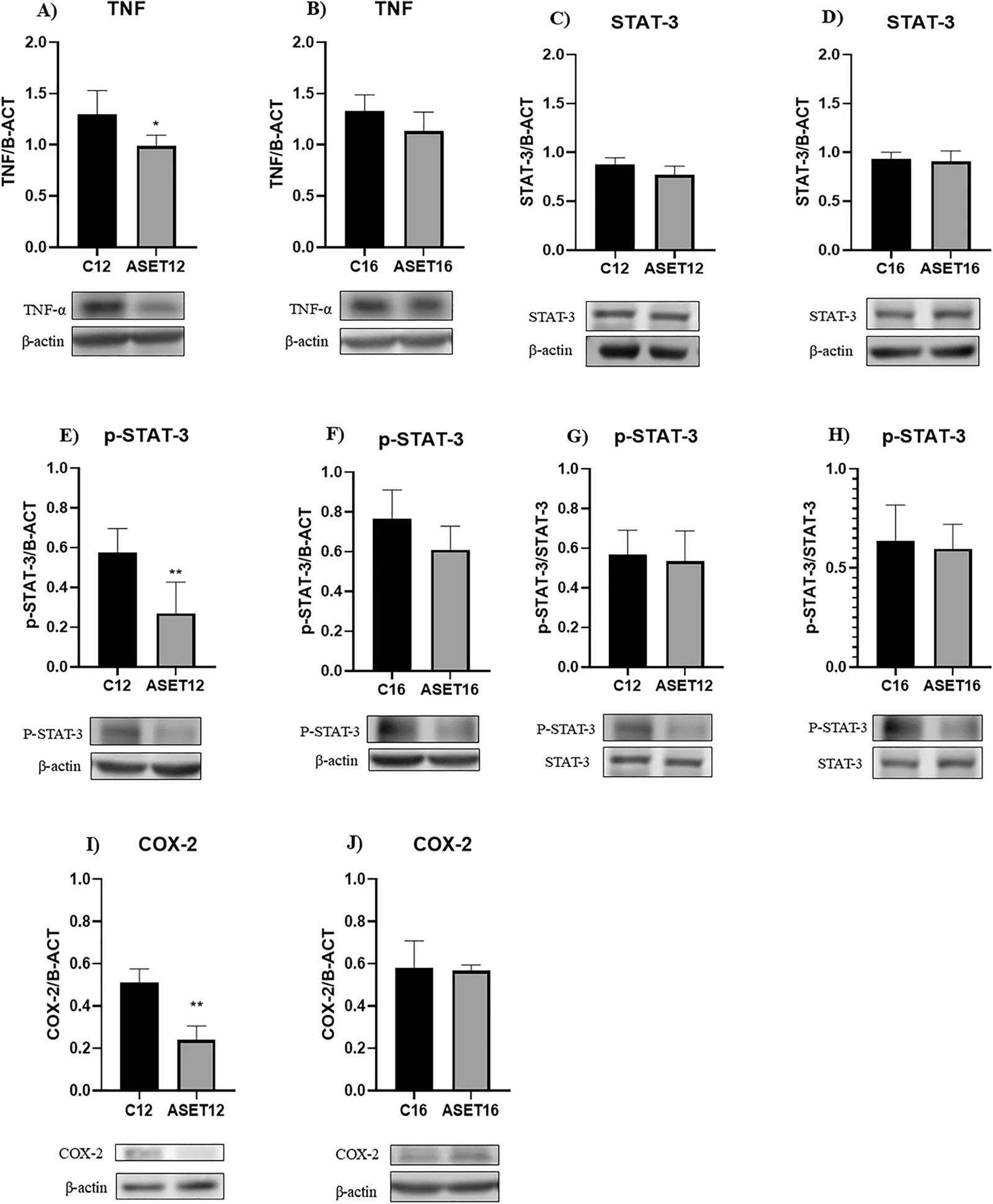
Protein levels of TNF (**A** and **B**), STAT3 (**C** and **D**), p-STAT3 (**E**–**H**), and COX-2 (**I** and **J**) in the ventral prostate lobe of TRAMP mice from the ASET12 and ASET16 groups and their respective controls, along with representative Western blotting bands. Statistical significance was considered between ASE-treated and untreated groups within the same experimental period (*p < 0.05; **p < 0.001)

In the ventral prostate lobe of the ASET12 group, a significant decrease (*p* < 0.05; *p* < 0.001) in protein levels of tumor necrosis factor-alpha (TNF-α) (Fig. 9A), phosphorylated signal transducer and activator of transcription 3 (p-STAT3) (Fig. 8E), and cyclooxygenase-2 (COX-2) (Fig. 9I) was observed in comparison to the C12 group.

Notably, in both ASET12 and ASET16 groups, when p-STAT3 was normalized to total STAT3 (its inactive form) as the endogenous control, no significant changes in protein levels were observed (Fig. 9G and H). In contrast, when normalized to β-actin as the endogenous marker, the ASET12 group exhibited a significant decrease (*p *< 0.001) in protein levels compared to the C12 group (Fig. 9E).

#### Modulation of the nuclear factor kappa B (NF-κB) pathway following ASE treatment

**Fig. 10:**
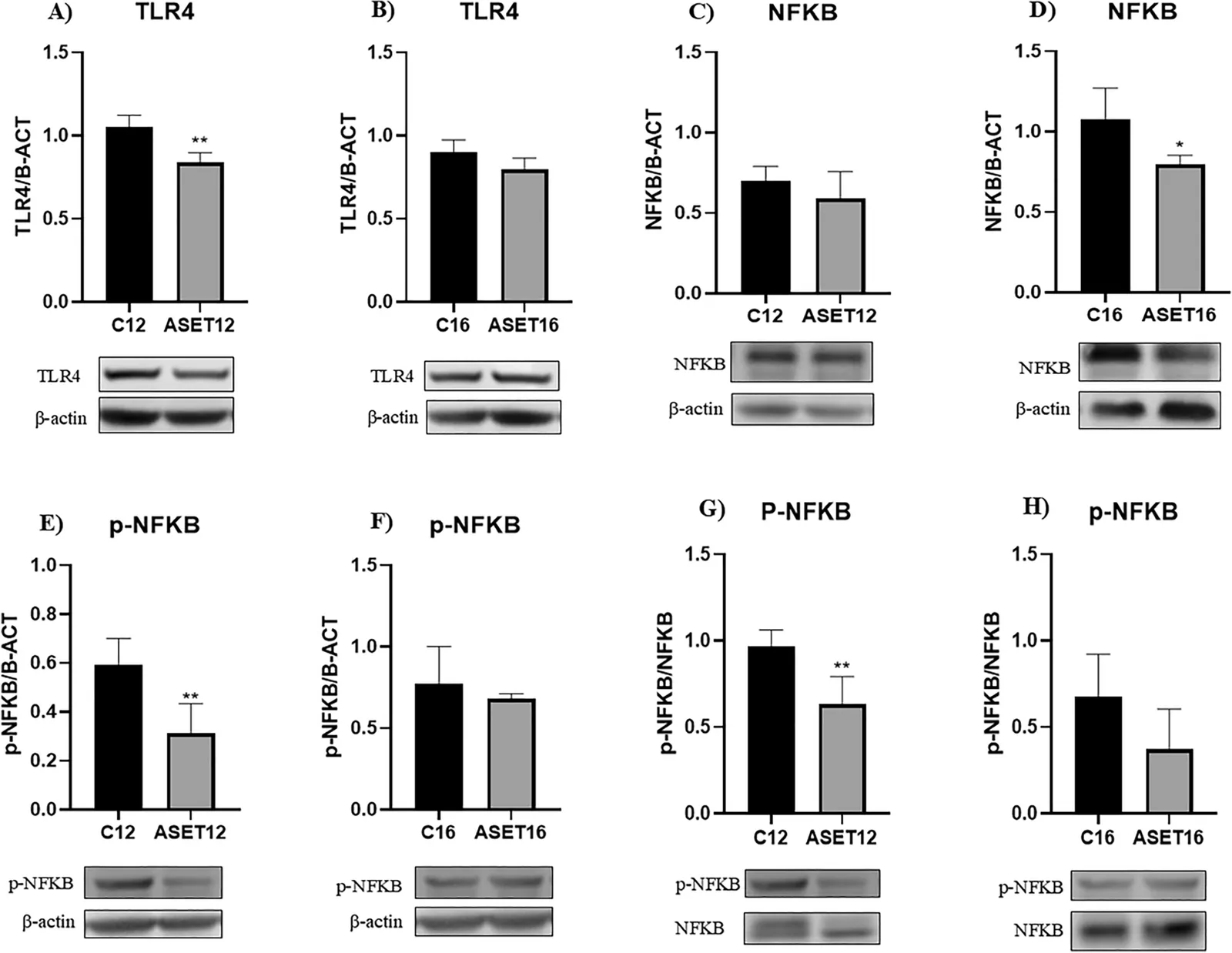
Protein levels of TLR4 (**A** and **B**), NFκB (**C** and **D**), and p-NFκB (**E**–**H**) in the ventral prostate lobe of TRAMP mice from the ASET12 and ASET16 groups and their respective controls, along with representative Western blotting bands. Statistical significance was considered between ASE-treated and untreated groups within the same experimental period (*p < 0.05; **p < 0.001)

In the ventral prostate lobe of the ASET12 group, a significant decrease (*p *< 0.01) in protein levels of Toll-like receptor 4 (TLR4) (Fig. 10A) and phosphorylated nuclear factor kappa B (p-NFκB) (Fig. 10E and G) was observed in comparison to the C12 group. In the ASET16 group, a significant reduction (*p *< 0.05) was observed only in total NFκB protein levels (Fig. 10D) compared to the C16 group.

It is noteworthy that, in the ASET12 group, when p-NFκB was normalized to both total NFκB (its inactive form/endogenous control) and β-actin, significant changes (*p *< 0.01) in protein levels were observed. These findings suggest that a potential modulation of the NFκB signaling pathway may be occurring in the ASET12 group.

### Modulation of cell proliferation markers, androgen receptor, and insulin-like growth factor 1 receptor by Araticum seed extract

**Fig. 11:**
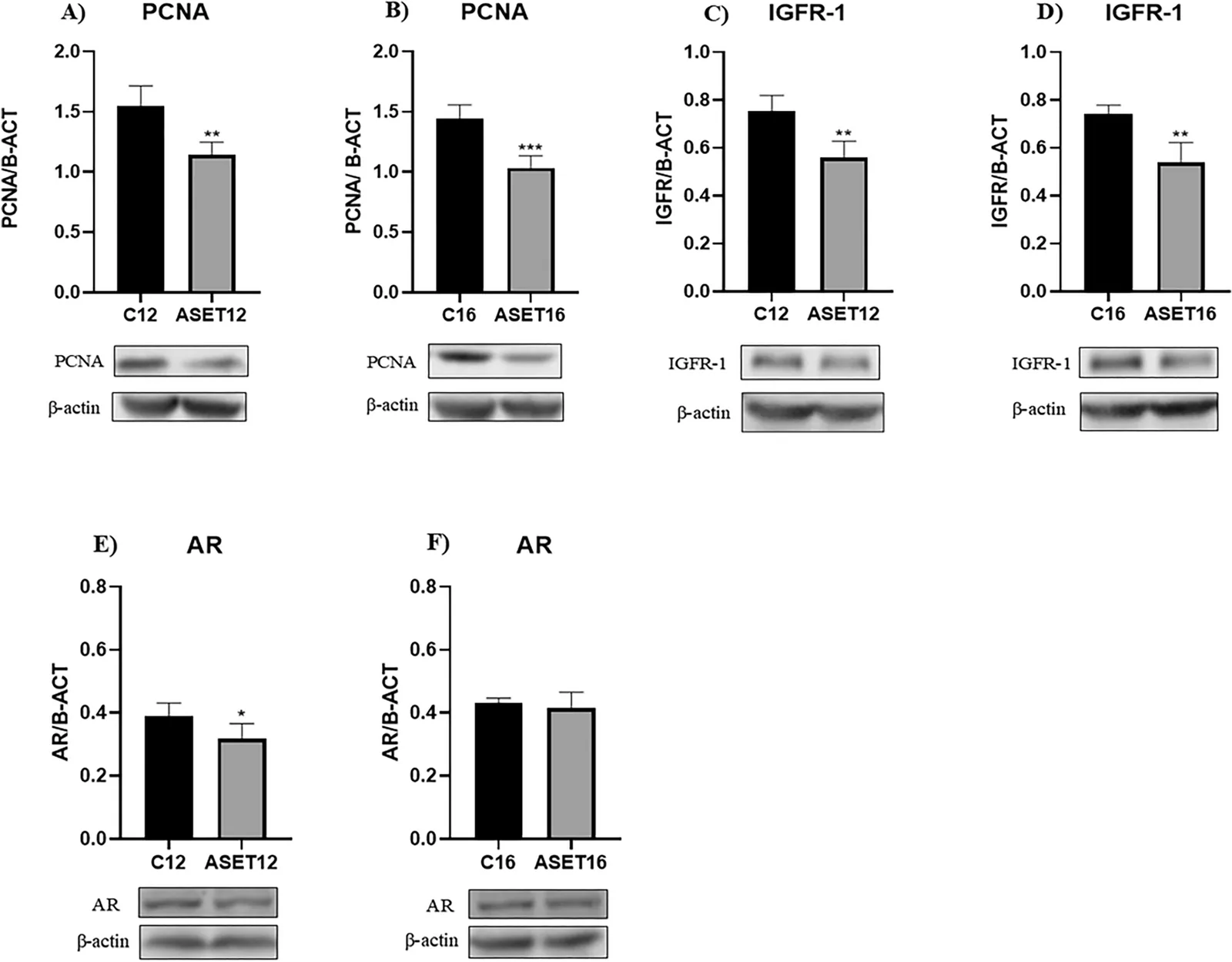
Protein levels of PCNA (**A** and **B**), IGFR-1 (**C** and **D**), and AR (**E** and **F**) in the ventral prostate lobe of TRAMP mice from the ASET12 and ASET16 groups and their respective controls, along with representative Western blotting bands. Statistical significance was considered between ASE-treated and untreated groups within the same experimental period (*p < 0.05; **p < 0.001; ***p < 0.0001)

In the ventral prostate lobe of the ASET12 and ASET16 groups, a significant decrease (*p *< 0.001; *p *< 0.0001) in protein levels of the proliferation marker PCNA and of the insulin-like growth factor 1 receptor (IGFR-1) was observed (Fig. 11A–D), compared to their respective control groups. However, a significant reduction (*p *< 0.05) in androgen receptor (AR) protein levels was observed only in the ASET12 group compared to the C12 group (Fig. 11E and F).

### ASE interference with mechanisms related to apoptotic cell death

**Fig. 12:**
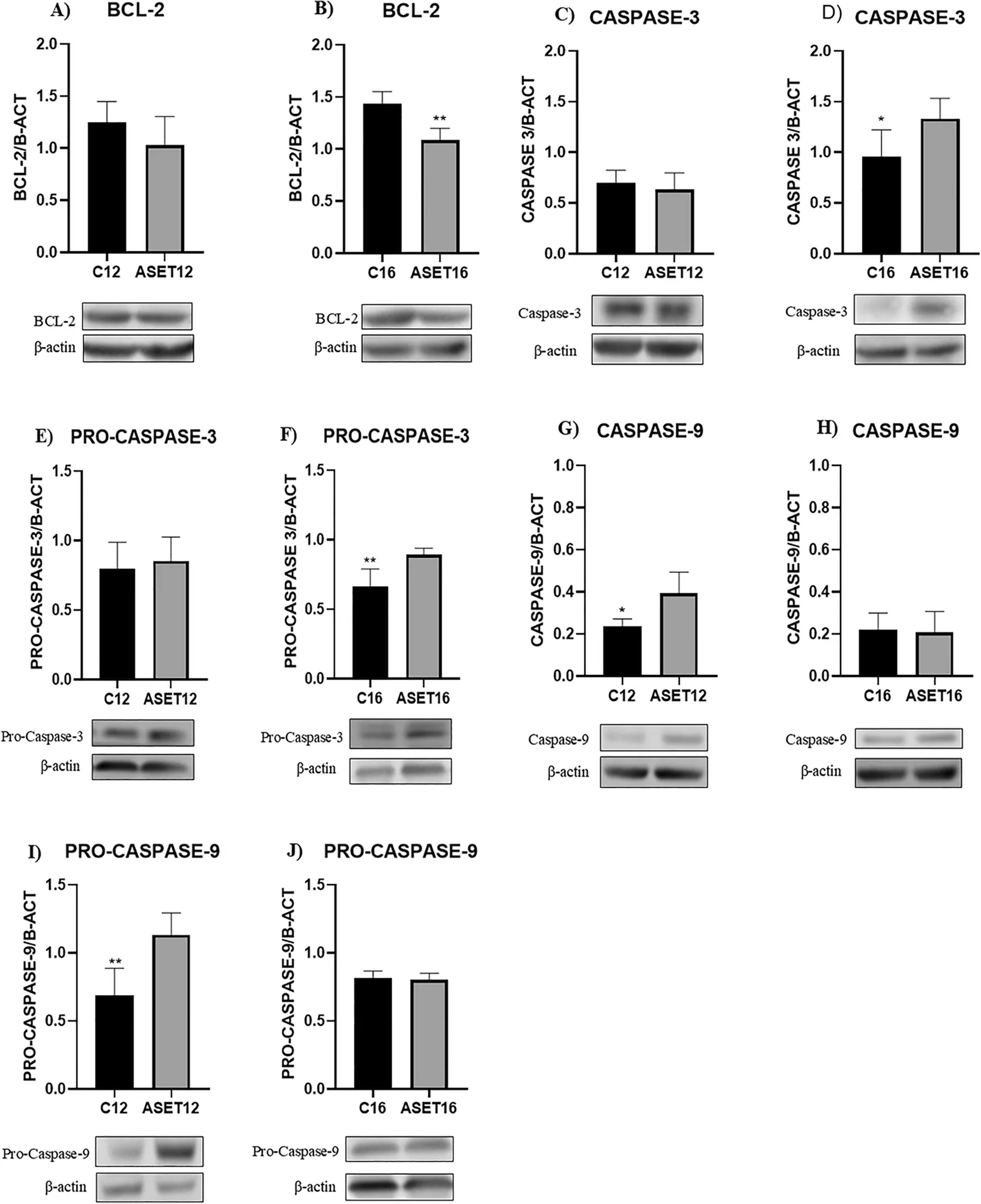
Protein levels of BCL-2 (**A** and **B**), CASPASE-3 (**C** and **D**), PRO-CASPASE-3 (**E** and **F**), CASPASE-9 (**G** and **H**), and PRO-CASPASE-9 (**I** and **J**) in the ventral prostate lobe of TRAMP mice from the ASET12 and ASET16 groups and their respective controls, along with representative protein blot bands. Statistical significance was assessed between ASE-treated and untreated groups within the same experimental period (*p < 0.05, **p < 0.001, ***p < 0.0001)

In the ventral prostate lobe of the ASET16 group, a significant decrease (*p* < 0.01) in the protein levels of the anti-apoptotic protein BCL-2 (Fig. 12B) was observed. In contrast, the protein levels of Caspase-3 and Pro-Caspase-3 significantly increased (*p *< 0.05; *p* < 0.01) (Fig. 12D and F) compared to the control group C16. In the ASET12 group, a significant increase (*p *< 0.05; *p *< 0.01) in the protein levels of Caspase-9 and Pro-Caspase-9 (Fig. 12G and I) was also observed in comparison to the C12 group.

## Discussion

The research identifies inflammation as a crucial factor in the development and progression of prostatic lesions due to cellular stress and repeated genomic damage (Kido et al. 2016; Mateus et al. 2019; Rossetto et al. 2023). Various experimental studies have focused on the inhibition or suppression of pro-inflammatory mediators as a therapeutic strategy for prostate cancer (Stark et al. 2015; Kido et al. 2016; Sharma et al. 2025). Rossetto et al. (2025) demonstrated that tempol (4-hydroxy-2,2,6,6- tetramethylpiperidine-1-oxyl) modulates genes and miRNAs related to the NFκB pathway in both in vitro and in vivo models of prostate cancer. Kido et al*.* (2016) reported that treatment of TRAMP mice with goniothalamin attenuated the pro-inflammatory response within the prostatic microenvironment, delaying prostate cancer progression. Similarly, treatment with celecoxib was shown to effectively regulate COX-2 expression in TRAMP mice, particularly at advanced stages of the disease. Mateus et al. (2019) also reported the efficacy of combining anti-inflammatory and antiangiogenic therapies in prostate cancer progression, indicating that celecoxib in combination with nintedanib represents an effective antitumor strategy against prostate cancer progression in TRAMP mice, while also preserving normal prostatic stroma.

Additionally, other studies using natural agents such as fruit extracts with potential anti-prostate cancer properties have been conducted by our research group in in vivo models. According to Longo et al. (2023), jabuticaba peel extract was able to interfere with hormonal signaling mediators, while Lamas et al. (2020) demonstrated that the patented jabuticaba peel extract (PJE) reduced levels of molecules related to oxidative stress, lipid peroxidation, inflammatory mediators, and CD3+ T cells, which were associated with the preservation of glandular morphological integrity in mice. Both studies attributed the anti-inflammatory effects in prostate cancer models to the wide variety of bioactive compounds present in jabuticaba peel extracts. According to Lemos et al. (2025), both the peel (APE) and seed (ASE) extracts of *Annona crassiflora Mart* reduced the viability of different prostate cancer cell lines and demonstrated pro-apoptotic and antiproliferative effects.

Currently, several compounds have been identified in *A. crassiflora* with potential chemopreventive and chemotherapeutic effects against cancer (Arruda et al. 2018; Prado et al. 2020). The characterization of the Araticum seed extract (ASE) by Lemos et al. (2025), indicated a phenolic profile similar to that reported by Arruda et al. (2018) and Prado et al. (2020), who identified classes of compounds such as tocopherols, phytosterols, acetogenins, rutin, and catechin. In the study by Lemos et al. (2025), the Araticum seed extract was found to have a high content of gallic acid, followed by catechin and quercetin, as well as strong antioxidant capacity. Gallic acid has been shown to induce apoptosis and inhibit proliferation in prostate cancer cells (Kaur et al. 2009; Jiang et al. 2022). Catechin decreased cell viability and induced apoptosis in prostate cancer models (Lin et al. 2021), a behavior similarly reported for quercetin, which, in addition to modulating androgen receptor activity, reduced prostate cancer cell viability and induced apoptosis (Ward et al. 2018). Therefore, to the best of our knowledge, the present study is the first to evaluate the chemopreventive and anti-inflammatory effects of *Annona crassiflora Mart* seed extract (ASE) in the TRAMP model of prostate cancer.

**Fig. 13:**
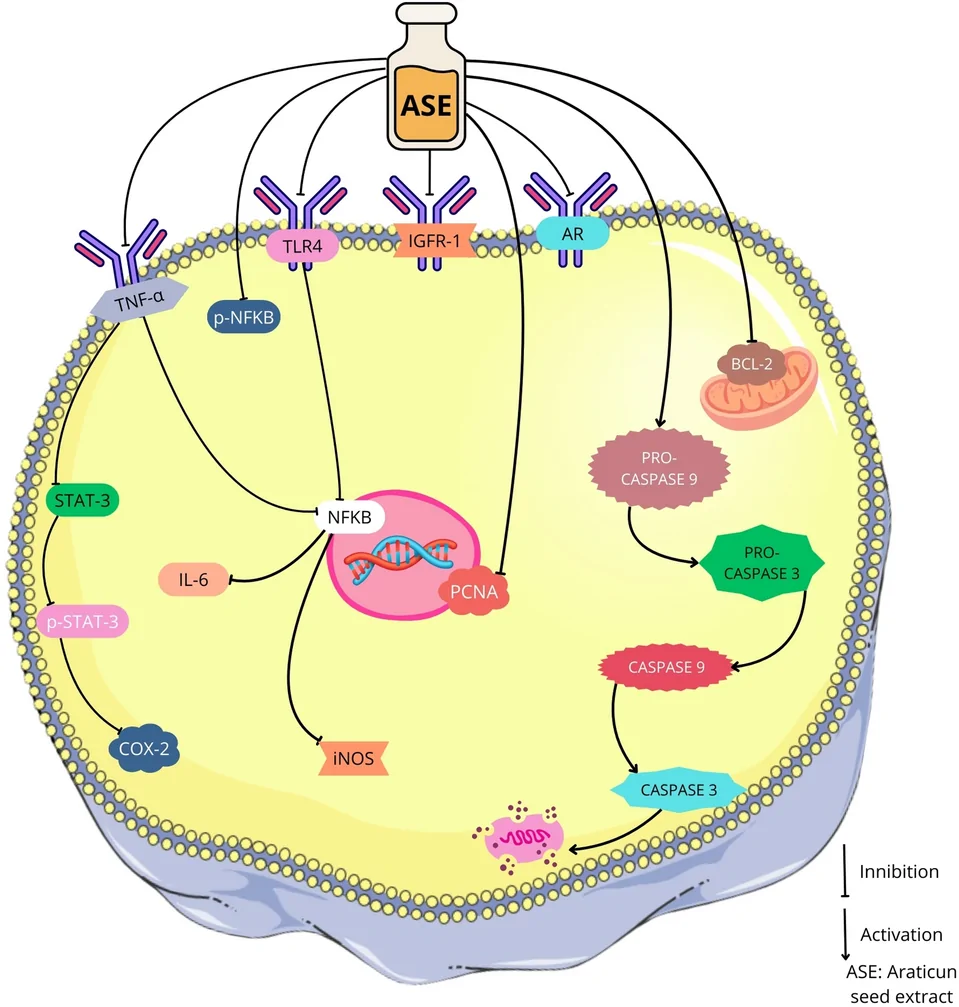
Anti-inflammatory, antiproliferative, and apoptotic mechanisms of ASE treatment in the ventral prostate lobe of TRAMP mice, highlighting the molecules evaluated in the present study. ASE inhibited early inflammatory signaling at the Toll-like receptor (TLR4), suppressed the initiation of the TNF-α inflammatory cascade, and subsequently downregulated p-STAT-3 and COX-2 expression. This led to the inhibition of the NFκB pathway, followed by a decrease in the pro-inflammatory interleukin IL-17 and inducible nitric oxide synthase (iNOS). ASE also inhibited the proliferative markers PCNA and IGFR-1, as well as the androgen receptor (AR). Additionally, ASE suppressed BCL-2 levels and activated pro-caspase-9, leading to the activation of caspase-9, followed by activation of pro-caspase-3 and caspase-3, ultimately inducing apoptosis. Abbreviations: ASE: *Annona crassiflora Mart* seed extract. Original illustration prepared by the authors using open-access images under a CC BY 4.0 license

In the present study, the anti-inflammatory effects of ASE were evaluated by analyzing different protein levels related to the NFκB signaling pathway. Following ASE treatment, two main mechanisms were observed in the ASET12 group. The first involved modulation of the early inflammatory signaling via the Toll-like receptor (TLR4) (Fig. 13), and the second was associated with the reduction or delay in the initiation of the TNF-α-driven inflammatory cascade. In contrast, in the ASET16 group, results indicated a downregulation solely in NFκB protein levels.

NFκB is a key transcription factor involved in the regulation of cell survival, inflammation, and cellular proliferation (Iksen et al. 2024). The literature reports to play a crucial role in the development of inflammation by regulating genes that encode pro-inflammatory cytokines, adhesion molecules, chemokines, growth factors, and inducible enzymes such as cyclooxygenase-2 (COX-2) and inducible nitric oxide synthase (iNOS) (Hanada and Yoshimura 2002). In cancer, NFκB remains constitutively active in the tumor microenvironment due to mutations in signaling molecules or in response to extracellular stimuli such as pattern recognition receptors (TLRs), receptors for pro-inflammatory cytokines (e.g., TNF-α), and antigen receptors (Hanada & Yoshimura 2002; Grivennikov & Karin 2010).

TLR4 plays a key role in the innate immune response (Han et al. 2015). It recognizes lipopolysaccharides from Gram-negative bacteria and other ligands. Upon activation, TLR4 initiates a signaling cascade that leads to the activation of NFκB (Han et al. 2015). Persistent NFκB activation results in the transcription of inflammatory genes and the production of pro-inflammatory cytokines (Hanada and Yoshimura 2002; Kashani et al. 2021), creating an inflammatory environment that favors tumor cell survival and proliferation (Marzo et al. 2007). In the current study, treatment with ASE led to a reduction in TLR4 and p-NFκB protein levels in the ASET12 group, resulting in decreased inflammation.

A second mechanism involves ASE’s ability to reduce or delay the onset of the inflammatory cascade mediated by TNF-α. Elevated TNF-α levels tend to increase inflammation and promote cancer cell survival and proliferation by activating the STAT-3 pathway, inducing the production of p-STAT-3, which in turn can upregulate COX-2 expression (Balkwill 2006; Galheigo 2015; Kashyap et al. 2021). In the present study, ASE’s anti-inflammatory effects were observed in the ASET12 group, which showed decreased protein levels of TNF-α, and consequently reduced levels of p-STAT-3 and COX-2 (Fig. 13). These findings are consistent with those of Laksmitawati et al. (2016) who demonstrated that *Annona muricata* (soursop) extracts like *Annona crassiflora Mart* (araticum) contain various classes of Annonaceous acetogenins (polyketide pathway metabolites), alkaloids, flavonoids, and sterols that confer antioxidant, anti-inflammatory, and anticancer properties (Rady et al. 2018). In that study, *A. muricata* extracts reduced TNF-α levels in a macrophage cell line (Laksmitawati et al. 2016). Therefore, the reduction in TNF-α and TLR4 protein levels at the early stage of cancer (ASET12) reinforces the potential of ASE as an eligible chemopreventive agent in prospective prostate cancer co-therapies, due to its suppressive effects on inflammation, particularly through regulation of the NFκB signaling cascade.

Furthermore, NFκB phosphorylation results in p-NFκB, the active form capable of binding to DNA and initiating target gene transcription (Hoesel and Schmid 2013). Notably, in the present results, in the ASET12 group, when p-NFκB was assessed alongside NFκB (inactive form) and β-actin as an endogenous marker, significant changes (*p *< 0.01) in protein levels were observed, suggesting possible regulation of the NFκB pathway in this group (Fig. 13), independent of the endogenous marker. This regulation further supports the anti-inflammatory potential of ASE and its consequent role in delaying prostate cancer progression.

ASE also led to a reduction in chronic inflammation. Chronic inflammation is characterized by the excessive expression of COX-2 in prostate cancer, leading to decreased prostate cell death (Hong et al. 2021).In the present study, COX-2 protein levels were significantly reduced in the early stage of cancer (ASET12). These results are in agreement with Hong et al. (2021), in which Wistar rats, treated with soursop stem bark extracts, showed a reduction in inflammation and hypertrophy in prostate tissues through the inhibition of COX-2, as well as inflammatory cytokines and growth factors. Additionally, Gupta et al. (2004) verified similar results to the results herein when treated TRAMP mice with celecoxib and, showed decreased COX-2 protein levels. Thus, we conclude that treatment with ASE was effective in mitigating the inflammatory process (preventing chronic inflammation) by reducing the COX-2 enzyme, particularly during the early response (ASET12).

The ASE anti-inflammatory activity was also evident in its effects on the iNOS and IL-17 inflammatory markers (Fig. 13), which are known to be involved in the transition from prostatic intraepithelial neoplasia to well-differentiated or poorly differentiated adenocarcinoma. iNOS plays a crucial role in tumor growth and angiogenesis and influences macrophage cytotoxicity and tumor-induced immunosuppression (Ferreira 2013). In the present study, ASE significantly decreased iNOS immunoreactivity in the ASET16 group. These findings are in agreement with those reported by Klotz et al. (1998), who detected iNOS expression in all histopathological samples from prostate carcinoma patients.

On the other hand, IL-17, has a pro-inflammatory role and it is primarily expressed by activated prostatic T lymphocytes (Djavan et al. 2009), It is produced in low amounts by healthy prostate cells, but in altered prostate tissue, its levels are increased, leading to an increase in the production of other members of the pro-inflammatory cytokine family, which is referred to as a local fine-tuner of the immune response (Djavan et al. 2009; Ferreira 2013). In the present study, IL-17 immunoreactivity was elevated in the C12 and C16 groups compared to the ASE-treated groups (ASET12 and ASET16), where a significant reduction in IL-17 immunoreactivity was observed. This suggests that ASE treatment exerted a protective effect on prostate tissue, reducing the transition from prostatic intraepithelial neoplasia to well-differentiated adenocarcinoma, thereby delaying prostate cancer progression.

These findings were further supported by the histopathological analysis conducted in this study, which revealed a lower incidence of adenocarcinoma in the ASE-treated groups, particularly in the early-stage group (ASET12). Additionally, ASE induced significant morphological changes in the ventral prostate lobe of TRAMP mice in both ASET12 and ASET16 groups, including a reduction in malignant prostatic lesions (HGPIN) and an increase in premalignant lesions (LGPIN). Notably, the ASET12 group also maintained healthy tissue architecture, reinforcing the evidence that ASE delayed prostate cancer progression.

Furthermore, ASE exhibited continued anti-inflammatory activity through the downregulation of the STAT-3 pathway and upregulation of BCL-2, along with a protective effect on prostatic tissue via activation of caspases (Fig. 13). This is particularly relevant given that STAT-3 promotes the expression of anti-apoptotic genes (*Bcl-2* and *Bcl-xL*) and regulates cell survival, with its active form, p-STAT-3, responsible for the transcription of genes that enhance inflammation and tumor growth (Yang et al. 2022; Bishop et al. 2014; Iksen et al. 2024). Thus, treatment with ASE resulted in decreased p-STAT-3 levels, particularly in the ASET12 group. Similar results were reported by Liang et al. (2011), who showed that the expression of Stat3 and p-Stat3 proteins was markedly reduced in prostate tumors of mice treated with si-Stat3 (a DNA vector-based Stat3-specific RNA) compared to tumors treated with PBS. Accordingly, the reduction in p-STAT-3 levels at the early stage (ASET12) contributed to the attenuation of the inflammatory process, thereby delaying prostate cancer progression.

Moreover, in the present findings, positive regulation of apoptotic mechanisms was observed through the reduction of BCL-2 in the ASET16 group. The upregulation of these apoptotic pathways may help control the proliferation of cancer cells, reduce tumor size, and prevent metastasis (Bray et al. 2009). BCL-2 is an anti-apoptotic gene involved in multiple stages of prostate cancer progression; its decrease can promote apoptosis in tumor cells (Brum and Brentani 2005; Galluzzi et al. 2018). Similar results were reported by Pieme et al*.* (2014), where *Annona muricata* extracts induced apoptosis and downregulated BCL-2 protein levels in human cancer cells (HL-60). Comparable outcomes were also documented by Moghadamtousi et al. (2015), who used *A. muricata* leaf extracts and observed pro-apoptotic effects in colon cancer cells, including BCL-2 downregulation and upregulating Bax, leading to cytochrome c release and subsequent activation of the caspase cascade, which is directly related to the triggering of apoptosis.

Thus, we suggest that the apoptotic mechanisms described above for *Annona muricata*, along with the findings of the present study, are consistent with the data reported by Jacobo-Herrera et al. (2019), which indicate that the compounds responsible for activating apoptosis are acetogenins, as they can inhibit complex I of the respiratory chain by binding to and blocking the NADH enzyme, thereby inhibiting ATP production and leading to cell death. As previously reported, acetogenins are more concentrated in the seeds of *A. crassiflora* than in the peel (Ramos et al. 2022). Therefore, it can be inferred that ASE exerts an effect on the regulation of apoptosis-related mechanisms.

The protective effect of ASE on prostatic tissue, through caspase activation, was observed at both treatment stages. Caspase-9 is an initiator caspase in the intrinsic apoptotic pathway, and its activation triggers a cascade of apoptotic events, culminating in the activation of executioner caspase-3 (Zhu et al. 2023). Caspase-3 is a critical executor of the caspase-dependent apoptosis cascade, responsible for chromatin condensation, DNA fragmentation, and ultimately, tumor cell apoptosis (Zhu et al. 2023). Pro-caspase-9 is the inactive precursor of caspase-9 that is activated during apoptosis, while pro-caspase-3 is the inactive form of caspase-3 that undergoes proteolytic cleavage to become active. This cleavage represents a key step in initiating the apoptotic process (Zhu et al. 2023). These markers are essential for understanding the balance between cell survival and death in prostate cancer and represent potential targets for therapies that aim to induce apoptosis in tumor cells (Kashyap et al. 2021).

In the present results, a significant increase in the protein levels of caspase-9 and pro-caspase-9 at the early stage of prostate cancer progression (ASET12 group) was observed, indicating the initiation of the apoptotic cascade, followed by a significant increase in caspase-3 and pro-caspase-3 at the final stage of prostate cancer progression (ASET16 group).

According to Yuan et al. (2003), annonacin belongs to a class of compounds (acetogenins) exclusively found in the *Annonaceae* family, which has also been identified in *A. crassiflora* (Mesquita et al. 2009). Annonacin from *Annona reticulata* seeds was used on T24 bladder cancer cells during the S phase, and it was observed that these cells were more vulnerable to annonacin cytotoxicity, with increased caspase−3 activity and apoptotic cell death. Yiallouris et al. (2018), also demonstrated that *A. muricata* leaf extract induced the expression of caspases 3 and 9 and inhibited cell proliferation in MIA PaCa-2 cells.

The presence of both pro-caspases is indicative of the potential initiation of apoptosis, and the increase in caspase-3 activity can accelerate the elimination of tumor cells (Bray et al. 2009; Hassan et al. 2020). Therefore, it can be suggested that ASE has a tissue-protective effect, maintaining the apoptotic process active from the early stage of prostate cancer (ASET12), as well as in the more advanced stage (ASET16).

In addition to its anti-inflammatory and pro-apoptotic effects, ASE also exhibited antiproliferative activity, evidenced by reduced protein levels of IGFR-1 and PCNA, as well as antiandrogenic effects, through decreased androgen receptor (AR) expression (Fig. 13). PCNA is a DNA-binding protein essential for replication during cell division, acting as a processivity factor for DNA polymerase and facilitating DNA synthesis (Zhao et al. 2011). The reduced PCNA protein levels observed at both treatment timepoints (ASET12 and ASET16) indicate a suppression of the proliferative process in the ventral prostate lobe. A similar antiproliferative effect was reported by Justino et al. (2021), where *A. crassiflora* bark extract decreased PCNA protein expression after 24h of treatment in human hepatocellular carcinoma (HepG2) cells. These histological findings are consistent with those of Asare et al. (2015), who showed that aqueous extract of *Annona muricata* leaves exhibited antiproliferative properties in BPH-1 cells and delayed tumor development in treated rats by inducing epithelial apoptosis and reducing prostate size. Yang et al. (2015), also observed that *A. muricata* leaf extract significantly inhibited tumor growth in human prostate cancer xenografts. These antiproliferative effects have been attributed to the complex phytochemical composition of the extracts, particularly acetogenins, also present in *A. crassiflora* seed extract, which demonstrated antiproliferative action in human prostate cancer PC-3 cells (Prado et al. 2020).

ASE’s antiproliferative effect on the proliferation marker PCNA was further confirmed by a significant reduction in PCNA immunoreactivity in both ASE-treated groups (ASET12 and ASET16). Our results are in agreement with the findings of Rasheed et al. (2023), who used an anthraquinone-derived non-steroidal anti-inflammatory drug, diacerein (DIA), as treatment and demonstrated a significant decrease in PCNA immunoreactivity in rats with BPH.

The insulin-like growth factor-1 receptor (IGFR-1) is a receptor that activates intracellular signaling pathways, promoting cell cycle progression and protecting cells from apoptosis. This contributes to tumor growth by enhancing cell proliferation and resistance to apoptosis (Fonseca 2017), and may play a role in the transition to androgen-independent disease. Inhibition of IGFR-1 signaling may retain AR in the cytoplasm and significantly alter androgen-regulated gene expression. Thus, the reduction in IGFR1 protein levels at both treatment ages (ASET12 and ASET16) suggests a link between the control of the proliferative process and androgenic regulation, as a significant decrease in AR protein levels was also observed in the early-stage prostate cancer group (ASET12).

AR is a nuclear receptor involved in early stages of prostate cancer by promoting cell proliferation and inhibiting apoptosis (Staal and Beyaert 2018). When bound to testosterone or other androgens, AR regulates the expression of genes involved in normal prostate development as well as progression toward castration-resistant prostate cancer (Ferreira 2011; Fontana et al. 2020). Notably, androgen deprivation or AR signaling blockade is associated with elevated inflammatory markers (Staal and Beyaert 2018). An in vivo study conducted by Taplin & Balk (2004) reported that when AR expression was suppressed, prostate cancer ceased to progress. In the study by Lamas et al*.* (2020), administration of jaboticaba peel extract (PJE) resulted in lower AR expression in the ventral prostate lobes of aged wild-type mice, thereby contributing to the reduction of proliferative epithelial alterations and stromal hypertrophy. These findings are in agreement with those obtained in the present study, in which AR levels were significantly reduced in the early-stage cancer group (ASET12), indicating the effectiveness of ASE treatment in slowing prostate cancer progression.

In summary, this study provides novel evidence that *Annona crassiflora* Mart. seed extract (ASE) exerts chemopreventive and pro-apoptotic effects in the TRAMP model of prostate carcinogenesis. ASE treatment modulated key molecular pathways related to inflammation, proliferation, and apoptosis, leading to reduced expression of proliferative and anti-apoptotic proteins and increased activation of caspases. These results support the hypothesis that bioactive compounds from araticum seeds, especially acetogenins, play a significant role in suppressing tumor progression and protecting healthy prostate tissue.

Despite the promising findings of the present study, some limitations should be considered. First, although the evaluation of systemic toxicity was limited to liver histopathology and body weight monitoring, without the inclusion of serum biochemical markers of liver function, the histological analyses consistently demonstrated preserved hepatic architecture, with no evidence of treatment-related morphological damage. Nevertheless, future studies should complement these findings by including biochemical and hematological parameters to provide a more comprehensive toxicological assessment. Another limitation is that only a single dose (100 mg/kg body weight) of *Annona crassiflora* seed extract was investigated. Although this prevented the establishment of a dose–response relationship, the selected dose was based on preliminary experiments and was sufficient to promote consistent biological effects across different levels of analysis. Significant reductions in inflammatory mediators, decreased cell proliferation, and attenuation of histopathological alterations associated with prostate cancer progression were consistently observed, supporting the biological activity of the extract at this concentration. Future investigations evaluating multiple doses and treatment regimens will be important to define the optimal therapeutic window and determine the minimum effective dose.

Another aspect that deserves consideration is that the mechanistic investigation focused primarily on inflammatory signaling pathways and cell proliferation, which represented the central objective of the present study. To address this objective, a comprehensive panel of complementary approaches was employed, including histopathological evaluation, immunohistochemistry, and Western blotting analysis of key proteins involved in inflammation and tumor progression. However, additional biological processes implicated in prostate cancer, such as epithelial–mesenchymal transition, angiogenesis, metastatic dissemination, oxidative stress, and immune cell infiltration, were not investigated. These analyses may contribute to a broader understanding of the molecular mechanisms underlying the protective effects of *A. crassiflora* seed extract and should be explored in future studies. Despite these limitations, the present work provides a comprehensive preclinical evaluation by integrating histopathological, immunohistochemical, and molecular analyses in a well-established model of prostate carcinogenesis. Future studies incorporating pharmacokinetic, toxicological, and functional analyses, together with clinical investigations, will be essential to further elucidate its mechanisms of action and therapeutic applicability in humans.

## Conclusion

ASE was shown to delay prostate proliferative progression, particularly at the early stage of prostate cancer (ASET12), by effectively modulating PCNA, IGFR-1, and AR expression. A significant decrease in the inflammatory process was identified through reduced IL-17 immunostaining and lower protein levels of biomarkers associated with the NFκB pathway, such as TLR4, TNF-α, p-STAT-3, p-NFκB, and COX-2. Furthermore, ASE demonstrated a protective effect on prostate tissue, as it was able to increase pro-apoptotic molecules such as Caspase-9 and Pro-Caspase-9. In the late stage of prostate cancer (ASET16), ASE treatment also influenced the balance between inflammatory and proliferative processes by reducing iNOS, IL-17, NFκB, PCNA, and IGFR-1 expression. Additionally, ASE maintained apoptotic activity and tissue-protective effects at this advanced stage by reducing the anti-apoptotic molecule BCL-2 and increasing pro-apoptotic markers Caspase-3 and Pro-Caspase-3.

Taken together, these findings suggest that ASE may have chemopreventive potential, contributing to delayed prostate cancer progression in the TRAMP model. ASE appears to act through anti-inflammatory and antiproliferative mechanisms, reinforcing the close relationship between inflammation and cellular proliferation, as it effectively regulated key biomarkers involved in these processes.

In conclusion, ASE demonstrated promising effects on inflammatory and apoptosis markers in TRAMP mice. As these are preclinical findings from a specific animal model, they cannot be directly extrapolated to humans. Further studies are needed to evaluate its safety, toxicity, and efficacy in additional models, and to explore its potential as a functional ingredient or nutraceutical for the prevention or management of prostate cancer.

## Data Availability

Data will be made available on request.

## Abbreviations

AR: Androgen receptor
BCL-2: B-cell lymphoma 2
PCa: Prostate cancer
COX-2: Cyclooxygenase-2
DMSO: Dimethyl sulfoxide
EDTA: Ethylenediaminetetraacetic acid
ES: Healthy epithelium
ESA: Araticum seed extract
GC: Coagulating gland
H&E: Hematoxylin and eosin
HGPIN: High-grade prostatic intraepithelial neoplasia
BPH: Benign prostatic hyperplasia
IGF: Insulin-like growth factor
IHC: Immunohistochemistry
IL: Interleukin
INCA: Brazilian national cancer institute
iNOS: Inducible nitric oxide synthase
LGPIN: Low-grade prostatic intraepithelial neoplasia
VP: Ventral prostate
NF-κB: Nuclear factor kappa B
LGPIN: Low-grade prostatic intraepithelial neoplasia
HGPIN: High-grade prostatic intraepithelial neoplasia
PCNA: Proliferating cell nuclear antigen
pNF-κB: Phosphorylated nuclear factor kappa B
PSA: Prostate-specific antigen
pSTAT3: Phosphorylated signal transducer and activator of transcription 3
STAT3: Signal transducer and activator of transcription 3
TGF: Transforming growth factor
TLR4: Toll-like receptor 4
TNF-α: Tumor necrosis factor-alpha
TRAMP: Transgenic adenocarcinoma of the mouse prostate
SV: Seminal vesicle
WB: Western blotting
WDA: Well-differentiated adenocarcinoma
